# CoCl_2_-induced alterations in antioxidative and inflammatory marker expression in an siRNA-based in vitro model of aniridia-associated limbal epithelial dysfunction

**DOI:** 10.1186/s12886-026-04982-8

**Published:** 2026-06-06

**Authors:** Shao-Lun Hsu, Nóra Szentmáry, Fabian N. Fries, Zhen Li, Ning Chai, Berthold Seitz, Maryam Amini, Shweta Suiwal, Tanja Stachon

**Affiliations:** 1 Dr. Rolf M. Schwiete Center for Limbal Stem Cell and Congenital Aniridia Research, Saarland University, Kirrberger Str. 100, 66424 Homburg/Saar, Germany; 2 Experimental Ophthalmology, Saarland University, Homburg/Saar, Germany; 3 Department of Ophthalmology, Saarland University Medical Center, Homburg/Saar, Germany

**Keywords:** Congenital aniridia, *PAX6* siRNA knockdown, Aniridia-associated keratopathy, CoCl₂ treatment, Inflammatory response

## Abstract

**Background:**

Congenital aniridia is a rare disease, accompanied by aniridia associated keratopathy (AAK) in most cases. Oxidative stress and inflammation are involved in the progression of AAK. We aimed to evaluate antioxidative and inflammatory gene and protein expression in a small interfering RNA (siRNA) *paired box 6 (PAX6)* knockdown limbal epithelial cell (LEC) model of aniridia under cobalt chloride (CoCl_2_)-induced stress.

**Methods:**

To mimic *PAX6* haploinsufficiency in congenital aniridia, *PAX6* expression was knocked down by 24-hour siRNA treatment in primary human LECs. Hypoxia-mimetic conditions were induced by 50 µM or 75 µM CoCl₂ for 48 h following siRNA transfection. Messenger RNA (mRNA) expression levels were analyzed by quantitative polymerase chain reaction (qPCR), while protein expression levels were assessed by enzyme-linked immunosorbent assay (ELISA) or western blotting.

**Results:**

Vascular endothelial growth factor A (VEGFA) protein levels were significantly increased in *PAX6* knockdown LECs compared with control siRNA-treated cells (*p* = 0.007). In contrast, *hypoxia-inducible factor 2-alpha* (*HIF-2α*) and *interleukin-6* (*IL-6*) mRNA levels (*p* = 0.031, *p* = 0.001), as well as interleukin-1 beta (IL-1β), IL-6, and interleukin-8 (IL-8) protein levels (*p* = 0.049, *p* < 0.001, *p* < 0.001, respectively), were significantly reduced in *PAX6* knockdown cells compared with control siRNA-treated LECs. CoCl₂ treatment (50 and 75 µM) reduced *hypoxia-inducible factor 1-alpha* (*HIF-1α*) mRNA expression in both groups (*p* = 0.019 and *p* = 0.007; *p* < 0.001 and *p* = 0.046, respectively). In control cells, 75 µM CoCl₂ increased *IL-1β* and *IL-8* mRNA expression (*p* = 0.022, *p* = 0.019) as well as IL-1β protein levels (*p* = 0.024), whereas IL-8 protein levels decreased at both concentrations (*p* = 0.002 and *p* < 0.001). No other analyzed genes showed significant expression changes in response to CoCl₂ treatment in either the control or *PAX6* knockdown groups.

**Conclusions:**

Our study demonstrates altered expression of hypoxia-related (*HIF-1α*,* HIF-2α*) and inflammatory (*IL-1β*,* IL-6*,* IL-8*) genes in response to CoCl₂ treatment or *PAX6* haploinsufficiency. Further investigation is needed to elucidate the effects of *PAX6* knockdown and its interaction with inflammatory pathways. This research may contribute to better understanding of congenital aniridia.

**Supplementary Information:**

The online version contains supplementary material available at 10.1186/s12886-026-04982-8.

## Introduction

Congenital aniridia is a rare disease according to the National Organization for Rare Diseases in the USA [1] with an estimated incidence rate of 1:64,000 to 1:100,000 [2]. It is known to be an autosomal dominant disease with high penetrance. In two-thirds of patients, there is inheritability whereas, one-third occurs sporadically [2]. The majority of congenital aniridia cases result from mutations in the *paired box 6* (*PAX6* gene), which are observed in approximately 90% of affected individuals [3]. These *PAX6*-associated cases are commonly referred to as classical aniridia [4]. The PAX6 protein is known to be a DNA-binding transcription factor that encodes for a downstream primary protein response in eye development. PAX6 is a key regulator of eye development. In the heterozygous *Pax6*^+/−^ small eye mouse model, *PAX6* haploinsufficiency results in cellular and tissue underdevelopment [5]. Aniridia commonly affects multiple ocular structures, including the iris and posterior segment, and has been associated with foveal, macular, and optic nerve head hypoplasia [2, 6]. The secondary complications include limbal stem cell deficiency, aniridia-associated keratopathy, lens ectopy, cataracts, secondary glaucoma, nystagmus, and chronic progressive dry eye disease. Clinical symptoms can develop in early childhood and lead to reduced visual acuity or even blindness [6].

One of the leading causes of vision loss in patients with aniridia is corneal opacity, commonly referred to as aniridia-associated keratopathy (AAK). AAK is characterized by the progressive loss of limbal stem cells and degeneration of the limbal palisades. Limbal stem cell deficiency, together with dysregulation of downstream genes controlled by *PAX6*, contributes to abnormal differentiation and impaired migration of corneal epithelial cells [6]. As a result, the damaged corneal epithelium becomes more susceptible to hypoxic stress and exhibits reduced resistance to reactive oxygen species (ROS) due to compromised antioxidative defense mechanisms [7]. Chronic oxidative stress and impaired wound healing further promote inflammation, scarring, and neovascularization of the corneal surface, thereby disrupting the corneal microenvironment and the limbal stem cell niche. These pathological processes have been directly linked to *PAX6* deficiency, as demonstrated in heterozygous *Pax6*^*+/−*^ mouse model of aniridia-associated keratopathy [7, 8].

**Fig. 1:**
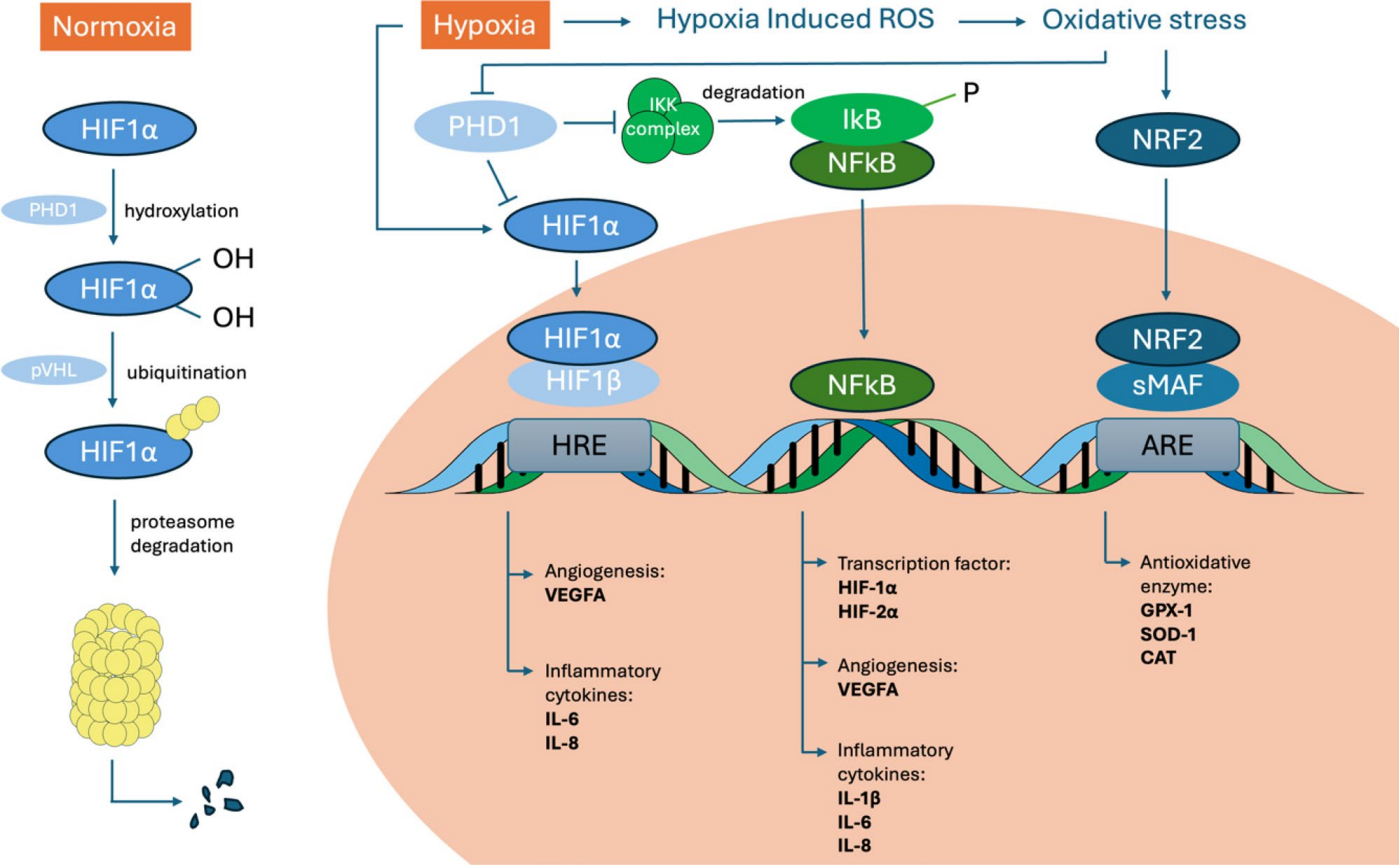
Cell response pathways under hypoxic and normoxic conditions. Under normoxic conditions, hypoxia-inducible factor 1α (HIF-1α) is continuously hydroxylated by prolyl hydroxylase domain protein 1 (PHD-1), leading to its ubiquitination and subsequent proteasomal degradation. Under hypoxic conditions, increased oxidative stress and reactive oxygen species (ROS) inhibit PHD1-mediated hydroxylation, resulting in stabilization and accumulation of HIF-1α. Stabilized HIF-1α translocates to the nucleus, binds to hypoxia response elements (HREs), and induces the transcription of downstream target genes that promote cellular adaptation to hypoxic stress. In parallel, additional transcription factors, including nuclear factor kappa-light-chain-enhancer of activated B cells (NF-κB) and nuclear factor erythroid 2-related factor 2 (NRF-2), are activated and regulate gene networks involved in angiogenesis, inflammation, metabolism, and antioxidative defense mechanisms

However, the molecular pathways and the pathomechanism of hypoxia-related chronic inflammation in *PAX6* haploinsufficiency are not known in detail. It is suggested that the cells tend to shift to different pathways for target gene transcription to better cope with hypoxic stress compared to normoxic conditions (Fig. 1). The downstream proteins usually account for inflammation, angiogenesis, or other transcription factors. It is known that hypoxia-inducible factor 1-alpha (HIF-1α) is inactivated under normoxic conditions through prolyl hydroxylase domain-containing protein 1 (PHD-1) hydroxylation, which makes hydroxylated HIF-1α targeted by Von Hippel–Lindau tumor suppressor (pVHL) for ubiquitination and proteasome degradation [9]. Under hypoxic conditions, inhibition of PHD-1 leads to the successful binding of HIF-1α and hypoxia-inducible factor 1-beta (HIF-1*β*) to transcript the hypoxia response element (HRE), which encodes for genes that modulate cell function under hypoxic stress [9, 10].

**Fig. 2:**
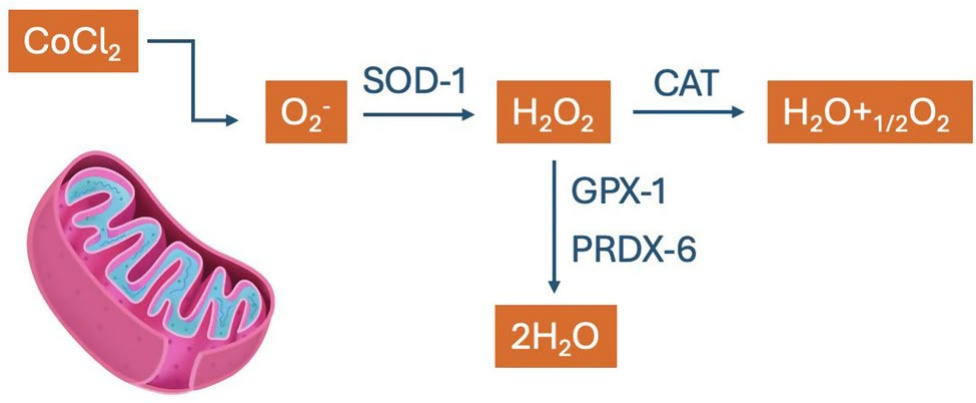
Endogenous enzymes in reactive oxygen species (ROS) metabolism serve as a defense mechanism. After being metabolized by the mitochondrial P450 enzyme, CoCl_2_ is converted to superoxide radicals (•O2). Subsequently, through an antioxidative pathway involving superoxide dismutase type 1 (SOD-1), catalase (CAT), glutathione peroxidase (GPX-1), and peroxiredoxin 6 (PRDX-6), these reactive oxygen species are converted to less harmful downstream products

Nuclear factor kappa-light-chain-enhancer of activated B cells (NF-kB) also serves as a transcriptional factor in the cells, under normoxic conditions. Transcription activity of NF-kB is inhibited by the nuclear factor of kappa light polypeptide gene enhancer in B-cells inhibitor (IkB) binding. The binding product is degraded by the IkappaB Kinase (IKK complex) through phosphorylation of IkB under hypoxic conditions. Additionally, inhibition of PHD-1 under hypoxia also leads to higher activity of the IKK complex, which allows NF-kB activation and gene transcription [11]. For nuclear factor erythroid 2-related factor 2 (NRF-2), the increased oxidative stress in the cell enhances NRF-2 activity. NRF-2 is translocated into the nucleus and dimerized with small Maf (sMAF) to the transcript for the antioxidant response elements (ARE). This complex thereby encodes for an antioxidative enzyme to further protect against ROS in the cell [12]. The accumulation of ROS under hypoxia leads to oxidative stress in the cells. Endogenous enzymes including superoxide dismutase type 1 (SOD-1), glutathione peroxidase 1 (GPX-1), catalase (CAT), and peroxiredoxin 6 (PRDX-6) are known as defense mechanisms to convert superoxide radicals to less harmful species (Fig. 2). SOD-1 catalyzes the dismutation of superoxide radical (•O2) to hydrogen peroxide (H_2_O_2_). H_2_O_2_ is rapidly converted to H_2_O and O_2_ by CAT. GPX-1 and PRDX-6 also neutralize H_2_O_2_ into H_2_O [13]. All pathways mentioned above contribute together against hypoxic stress in the cells. In congenital aniridia, corneal limbal epithelial cells are exposed to chronic hypoxic stress, which may be associated with a persistently inflammatory microenvironment. This sustained inflammation is hypothesized to contribute to the progressive limbal stem cell deficiency observed in AAK [14]. However, little is known about the role of the *PAX6* gene in the regulatory pathways of chronic hypoxia and its downstream impact.

CoCl₂ is widely used to establish in vitro hypoxia models [15], primarily acting through ROS generation via mitochondrial cytochrome P450 enzymes [16]. Although CoCl₂ does not fully replicate physiological hypoxia, it was used in the present study as a practical trigger to evaluate antioxidative and inflammatory gene and protein expression levels in the small interfering RNA (siRNA) *PAX6* aniridia limbal epithelial cell model.

## Materials and methods

### Cell culture

The project was approved by the Ethical Committee of Saarland, Germany (178/22). All procedures were conducted in accordance with the principles of the Declaration of Helsinki. Written informed consent was obtained from all participants. Thirteen human corneoscleral rings (age: 80.25 ± 8.44 years [61–90], median: 83.5 years; 5 [55.56%] males) were obtained from the LIONS Cornea Bank Saar-Lor-Lux, Trier/Westpfalz (Table 1). Ethical approval was granted under a broader research protocol on aniridia-associated keratopathy. This study utilized residual corneoscleral ring tissue from standard corneal transplantation procedures, which falls within the scope of the approved protocol.

**Table 1:** Detailed information on corneal donor tissue

Donor number	Gender	Age (years)
1	n/a	83
2	n/a	n/a
3	n/a	73
4	male	61
5	n/a	83
6	female	90
7	male	74
8	female	84
9	female	84
10	male	85
11	female	88
12	male	72
13	male	86

Primary human limbal epithelial cells from 13 donors (age: 80.25 ± 8.44 years [61–90], median: 83.5 years; 5 [55.56%] males) were used. Donors 1–5 were used for the viability assay (XTT assay) (*n* = 5 biological replicates, 2 technical replicates each), these donors were independent from those used in Quantitative PCR (qPCR), Western blot (WB), and enzyme-linked immunosorbent assay (ELISA) experiments due to limited cell availability. Donors 6–13 were used for qPCR (*n* = 7 biological replicates, 2 technical replicates each), Western blotting (*n* = 8 biological replicates, 1 technical replicate each) and ELISA (*n* = 6 biological replicates, 2 technical replicate each). qPCR, Western blot, and ELISA were performed using the same donor pool with assay-specific subsets depending on sample availability. n/a: not available

To isolate primary human limbal epithelial cells (LECs), the limbal region was punched out under light microscopy to obtain the upper half of the limbal depth and placed in keratinocyte growth medium (KGM3, PromoCell GmbH, Heidelberg, Germany) with collagenase A (1 mg/ml) (Roche Diagnostic GmbH, Mannheim, Germany, No. 10103578001) at 37 °C for 24 h. The KGM3 Medium was supplemented with a Supplement Mix and CaCl_2_ solution, provided by the manufacturer. This medium will be referred in the following text as KGM3. The lysed tissue was then detached using trypsin and ethylenediaminetetraacetic acid (trypsin-EDTA) (0.05% trypsin/0.02% EDTA, Sigma-Aldrich^®^ GmbH, Geisenheim, Germany), and the suspension was centrifuged at 800 x g for 4–5 min at room temperature. The resulting cell pellet was resuspended in KGM3 and seeded into a 6-well culture plate, with 2.5 ml of KGM3 per well.

Cell cultures were maintained with medium change every 2–3 days in an incubator at 37 °C with 95% relative humidity and 5% CO_2_ atmosphere. Trypsinization was used to remove limbal fibroblasts (> 95% limbal epithelial cells). The cells were cultured until reaching 80% confluence in 6 wells. Only primary LECs between P2-P4 with desirable confluence were included in the experiments.

### Viability assay

For the methoxynitrosulfophenyl-tetrazolium carboxanilide assay (XTT assay) (Roche, Cell Proliferation Kit II, XTT). XTT assay included 5 independent biological replicates, each with 2 technical replicates that were averaged prior to analysis (Table 1). These donors were independent from those used in qPCR, Western blot, and ELISA experiments due to limited cell availability. We used a 96-well plate with 10,000 cells/ cm² cell density and LECs were cultured until reaching 90% confluence.

The CoCl_2_ stock solution was prepared from CoCl_2_ powder (PHD inhibitor, Sigma-Aldrich^®^ GmbH, Geisenheim, Germany, No. 60818) diluted in KGM3 to achieve the following concentrations: 0µM, 50µM, 75µM, 150µM, 300µM, 1000µM. For each well, corneal epithelial cells were cultured using 100µL of different concentrations of the CoCl_2_ solution for 48 h, and an XTT assay was used to measure cell viability. 50µL XTT labeling reagent and 1µL electron coupling reagent (Roche, Cell Proliferation Kit II, XTT) were loaded into each well, and the optical density (OD) value was measured by the Tecan Infinite F50 Absorbance Microplate Reader (Tecan Group AG, Männedorf, Switzerland). Cell viability data were analyzed using repeated-measures one-way ANOVA followed by Dunnett’s multiple-comparison test.

### siRNA transfection

**Fig. 3:**
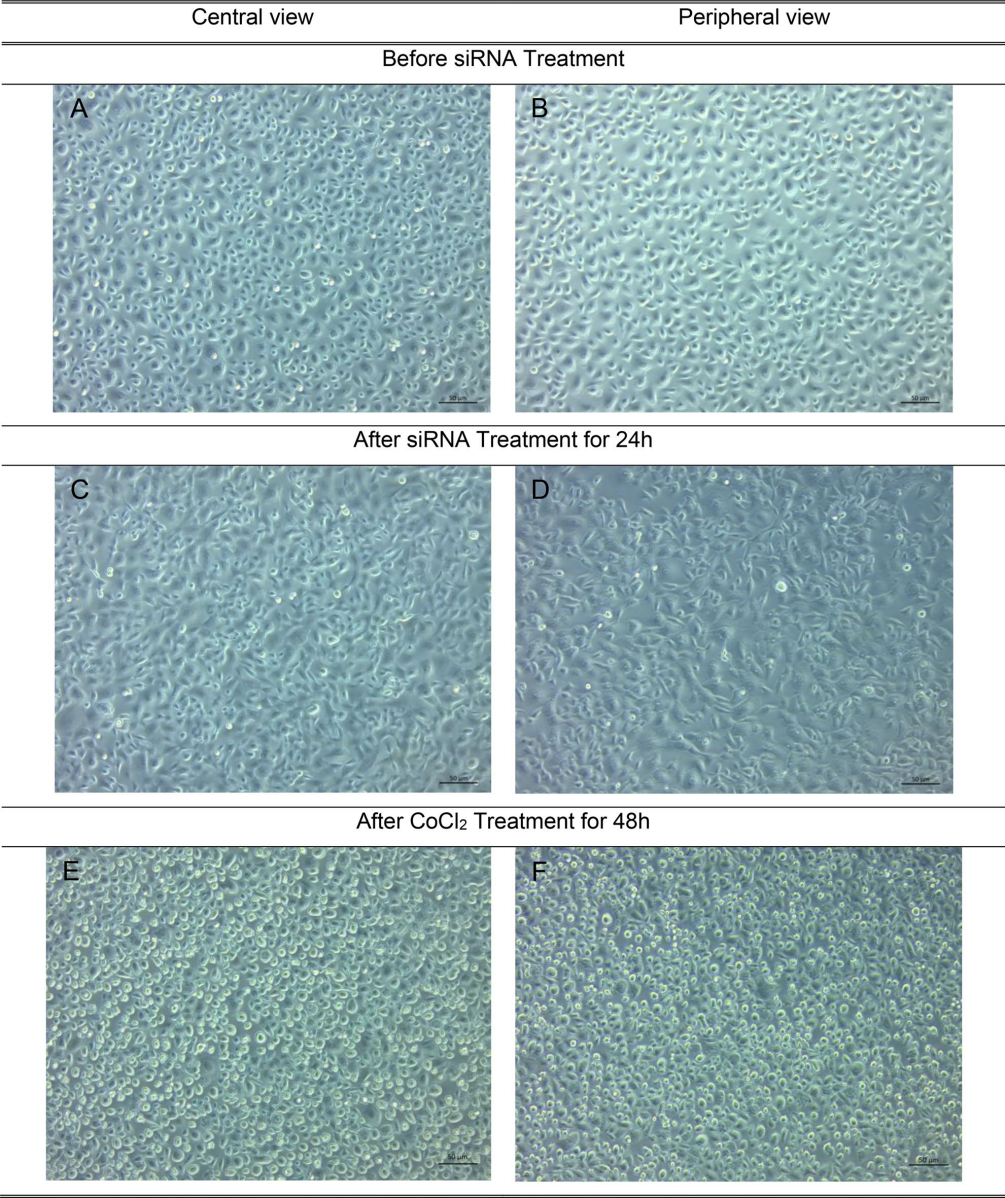
Limbal epithelial cell (LECs) morphology before and after siRNA and CoCl_2_ treatment. According to our protocol, siRNA treatment starts once the cells reach 80% confluence in both central and peripheral areas of well. Only pure LECs populations between passages 2 and 4 (P2-P4) are included. Cells before siRNA treatment present a small round shape and a high nuclear/cytoplasmic (N/C) ratio, indicating a high replication rate under healthy conditions (**A, B**). After 24 h of siRNA treatment, cell confluence decreases, with a more irregular cell size and cell borders (**C, D**). The cells recover following culture medium change (keratinocyte growth medium), 24 h after siRNA treatment. Finally, 48 h after CoCl_2_ treatment, the LECs are harvested. At this stage, LECs present with large granule formation in the cell body, indicating the accumulation of glycogen or lipids under CoCl₂-induced stress (**E, F**)

*PAX6* expression was knocked down using 5 nM siRNA (Silencer Select Negative Control Catalog #4390843, Silencer Select siRNA *PAX6* Catalog #4392420; Thermo Fisher Scientific) in combination with Lipofectamine 2000 transfection reagent (0.75 µL per 3 mL medium per well in a six-well plate). This pre-designed siRNA is designed to target sequences shared across known splice variants and is therefore expected to reduce the expression of major *PAX6* isoforms, including canonical *PAX6* and *PAX6(5a)*. Transfection was performed for 24 h once cells reached approximately 80% confluence to mimic *PAX6* haploinsufficiency observed in congenital aniridia (Fig. 3). The control group was treated with scrambled siRNA (Negative Control #1 siRNA). Cells were transfected with siRNA for 24 h, after which the culture medium was replaced. Following an additional 24-hour recovery period in fresh medium, cells were treated with cobalt chloride (CoCl₂; prolyl hydroxylase domain inhibitor; Sigma-Aldrich^®^ GmbH, Taufkirchen, Germany, No. 60818). CoCl₂ was applied at concentrations of 0, 50, or 75 µM for 48 h in both the *PAX6* knockdown and scrambled siRNA control groups.

Thereafter, the cell culture supernatants were collected for subsequent analysis and the cell monolayer was rinsed with Dulbecco’s Phosphate-Buffered Saline (DPBS) (Sigma-Aldrich, Dulbecco’s Phosphate-Buffered Saline). The cells were then harvested using SKP Lysis Buffer (NORGEN RNA/DNA/Protein Purification Plus Kit) mixed with 2-Mercaptoethanol. The samples were stored at -80 °C for further use.

### RNA/ protein isolation and measurement

The ribonucleic acid (RNA) and protein were isolated according to the instructions provided by the manufacturer (NORGEN RNA/DNA/Protein Purification Plus Kit, No. 47700-NB, BioCat GmbH, Heidelberg, Germany). The protein was quantified through Bradford’s method. The bovine serum albumin served as the standard protein and the absorbance was measured at 595 nm by Tecan Infinite F50 Absorbance Microplate Reader (Tecan Group AG, Männedorf, Switzerland). The diluted RNA was quantified with a UV/VIS spectrophotometer (Analytik Jena AG, Jena, Germany). For complementary deoxyribonucleic acid (cDNA) synthesis (OneTaq^®^ RT-PCR Kit (New England Biolabs Inc., Ipswich, USA), 500 ng RNA was used.

### Quantitative PCR

Quantitative PCR (qPCR) was conducted using primers from Qiagen GmbH (Hilden, Germany) (Table 2) and AceQ^®^ qPCR SYBR Green Master Mix (Vazyme Biotech, Nanjing, China). The PCR Thermocycler QuantStudio 5 Real-Time PCR System (ThermoFisher ScientificTM GmbH, Dreieich, Germany) was used. The amplification protocol was set at 95 °C (10 s), 64 °C (10 s), and 72 °C (45 s) for 40 cycles. The measured cycle threshold (Ct) values were normalized to the average of TATA-binding protein (TBP) and β-glucuronidase (GUSB) to yield ΔCt (ΔCt= individual Ct value - Ct mean of TBP/GUSB) and the ΔΔCt was defined as the ΔCt mean - individual ΔCt. The geometric mean was calculated accordingly as the fold change of ΔΔCt (geometric mean = 2^ ΔΔCt) to demonstrate the difference between the Ct value of target samples compared to the Ct mean of the reference gene, in a square multiple manner. qPCR experiments included 7 independent biological replicates, each with 2 technical replicates that were averaged prior to analysis. qPCR, Western blot, and ELISA were performed using the same donor pool with assay-specific subsets depending on sample availability (Table 1).

**Table 2:** Primer pairs used for qPCR

Name of the primer	Qiagen Cat. No	Amplicon size(bp)
CAT	QT00079674	60
GPX-1	QT00203392	105
GUSB	QT00046046	96
HIF-1α	QT00083664	104
HIF-2α	QT00069587	127
IL-1β	QT00021385	117
IL-6	QT00083720	107
IL-8	QT00000322	102
NF-kB	QT02324308	136
NRF-2	QT00027384	153
PAX6	QT00071169	113
PHD-1	QT00222684	123
PRDX-6	QT00000098	139
SOD-1	QT01008651	66
TBP	QT00000721	132
VEGFA	QT01010184	273, 222, 204, 150*

*VEGFA primer is a mix of NM_001025366 (transcript variant 1), NM_001171629 (transcript variant 7), NM_001171623(transcript variant 1), NM_001171625(transcript variant 3), NM_001025368(transcript variant 4), NM_001033756(transcript variant 7), NM_001025367(transcript variant 3), NM_001171624(transcript variant 2), NM_003376(transcript variant 2), NM_001171626(transcript variant 4), NM_001287044(transcript variant 10)

### Western blot analysis

20 µg protein from each sample was dyed with 5µL Laemmli Sample Buffer (Bio-Rad Laboratories, Hercules, California, USA) and was heated up for 5 min at 95 °C. 1 µL Precision Plus Protein Dual Color Standards (BIO-RAD) reagent was used as a marker. The proteins were subsequently loaded into NuPAGE™ bis-tris precast 4–12% bis- tris gels (ThermoFisher Scientific™ GmbH, Dreieich, Germany) for electrophoresis, and were transferred onto a nitrocellulose membrane using Trans-Blot^®^ Turbo™ Transfer System (Bio-Rad Laboratories, Hercules, California, USA) afterward. The blot was labeled with Invitrogen™ No-Stain™ Protein Labeling Reagent (Thermo Fisher Scientific, Waltham, USA) to acquire the total protein amount for normalization. Antibodies for the markers of interest were purchased from Cell Signaling Technology (Frankfurt am Main, Germany) (Table 3), and were diluted in Western Froxx Solution B (anti-rabbit HRP) (neo Froxx GmbH, Einhausen, Germany) for use. The Western Lightning^®^ Plus ECL Reagent (PerkinElmer, Inc., Waltham, USA) was used for the immunolabelling of the target protein. Blots were stripped in stripping buffer (Bio Froxx) and were washed with Western Froxx Wash Solution (neo Froxx GmbH, Einhausen, Germany), if necessary. The protein was quantified and normalized through the iBright Imaging system and was analyzed by iBright™ Analysis Software with total protein normalization (TPN) and local background correction. Western blot analyses included 8 independent biological replicates, each with 1 technical replicate. qPCR, Western blot, and ELISA were performed using the same donor pool with assay-specific subsets depending on sample availability (Table 1).

**Table 3:** Antibodies used for western blot analysis

Antibody	Referred as	Catalog number	Dilution	Molecular weight
NF-kB p65 (D14E12) XP Rabbit mAb	NF-kB	#8242	1:1000	65 kDa
Anti-PAX-6 rabbit Ab	PAX6	#AB2237	1:1000	48 kDa
PHD-1 Polyclonal Antibody	PHD-1	#PA5-96102	1:1000	40 kDa
PRDX-6 (D9J9H) Rabbit mAb	PRDX-6	#64,329	1:1000	25 kDa
SOD-1 Rabbit Ab	SOD-1	#2770	1:1000	18 kDa
VEGFA Rabbit Polyclonal Antibody	VEGFA	#19003-1-AP	1:1000	36 kDa

### ELISA

Cytokine concentration in the supernatant was assessed using ELISA. Interleukin-1β (IL-1β), interleukin 6 (IL-6), interleukin 8 (IL-8), and vascular endothelial growth factor A (VEGFA) protein expression levels were measured according to the protocol provided by DuoSet^®^ ELISA Kits from R&D Systems Europe, Ltd. (Abingdon, UK). The measurement was performed by Tecan Infinite F50 Absorbance Microplate Reader (Tecan Group AG, Männedorf, Switzerland) at 450 nm wavelength. ELISA experiments included 6 independent biological replicates, each with 2 technical replicates that were averaged prior to analysis. qPCR, Western blot, and ELISA were performed using the same donor pool with assay-specific subsets depending on sample availability (Table 1). The result was normalized to the total protein amount, acquired from the Bradford test for further statistical analysis.

### Statistical analysis

The GraphPad Prism 10.0 was used for statistical analysis. Normality was assessed using the Shapiro–Wilk test. For data with a normal distribution, a two-way ANOVA followed by multiple comparisons using the Dunnett method was performed to evaluate differences between groups. A repeated-measures two-way ANOVA was used, with donor included as a repeated factor to account for pairing between control and *PAX6*-knockdown conditions. Outliers were excluded using Grubbs’ test. Results were normalized to the average of the control group and expressed as percentages. A p-values below 0.05 were considered statistically significant. mRNA results were visualized with geometric mean±geometric standard deviation (SD), and protein results are shown as mean±standard deviation.

## Results

### Cell viability after 48 h CoCl_2_ treatment

**Fig. 4:**
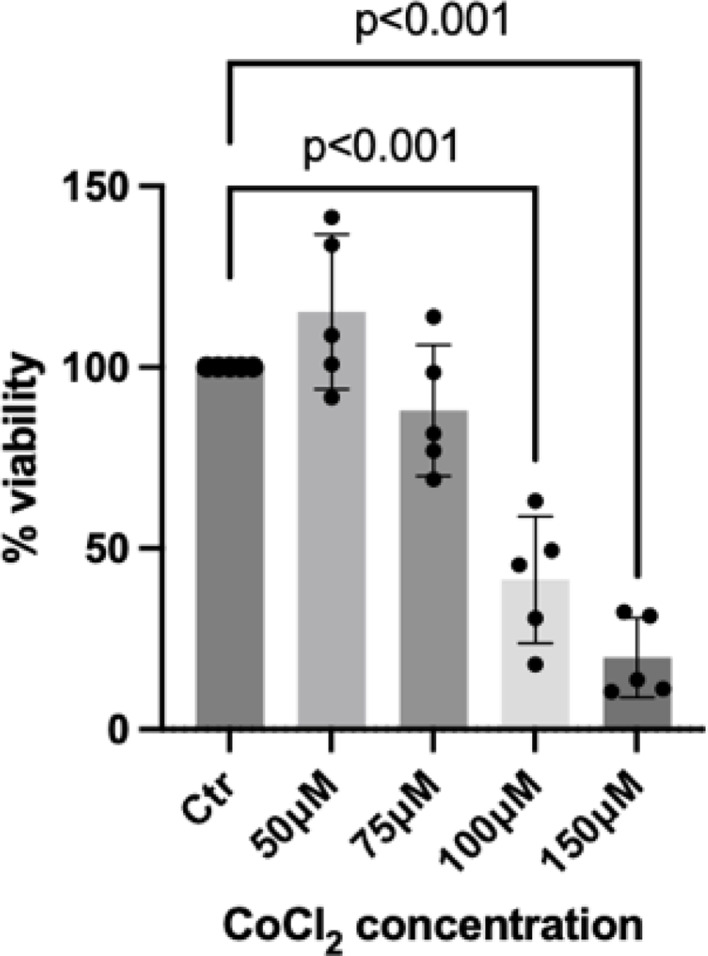
Cell viability of limbal epithelial cells after CoCl_2_ treatment, measured by XTT assay (*n* = 5 biological replicates, 2 technical replicates each). The 5 donors used in XTT assay were independent from those used in qPCR, Western blot, and ELISA experiments due to limited cell availability Table 1). Viability is expressed as percentage values and presented as geometric mean ± standard deviation (SD). Cell viability data were analyzed using repeated-measures one-way ANOVA followed by Dunnett’s multiple-comparison test. A repeated-measures design was used to account for donor pairing. Outliers were excluded using Grubbs’ test. Statistically significant p values (< 0.05) are highlighted. Following 48-hour CoCl_2_ treatment mimicking hypoxia, limbal epithelial cell viability decreased significantly from 100 µM CoCl_2_ concentration (*p* < 0.001)

After 48 h of CoCl_2_ treatment at 100 µM concentration, limbal epithelial cell viability significantly decreased compared to controls (*p* < 0.001) (Fig. 4). Hence, 50µM and 75µM CoCl_2_ concentrations were used as stimuli in subsequent experiments.

### PAX6 mRNA and protein levels following siRNA knockdown and CoCl_2_ treatment

**Fig. 5:**
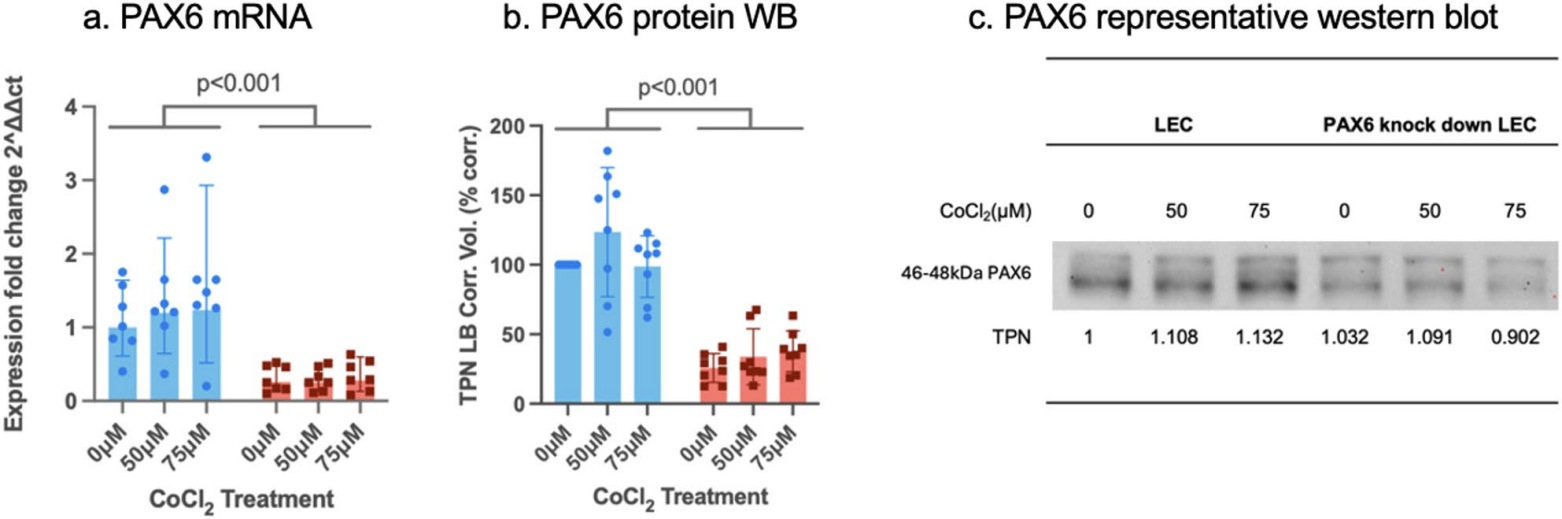
Paired box 6 (PAX6) mRNA (**a**) and protein (**b, c**) levels in control siRNA (blue bars) and in PAX6 knockdown (red bars) limbal epithelial cells (LECs) without and after 50µM or 75µM CoCl_2_ incubation for 48 h. Quantitative PCR (qPCR; *n* = 7 biological replicates, 2 technical replicates each) and Western blotting (WB; *n* = 8 biological replicates, 1 technical replicate each) were performed. qPCR, Western blot, and ELISA were performed using the same donor pool with assay-specific subsets depending on sample availability (Table 1). mRNA values are shown as geometric mean±geometric standard deviation (SD). Total protein staining was used for protein normalization of each lane. The total protein normalization factor (TPN) is indicated below each lane. Band intensity from WB is shown with mean ± SD. Two-way ANOVA, followed by Dunnett’s test was used. A repeated-measures design was used to account for donor pairing. Outliers were excluded using Grubbs’ test. Significant p values (< 0.05) are highlighted PAX6 mRNA (**a**) and protein levels (**b**) were significantly lower after *PAX6* siRNA knockdown, than using control siRNA (*p* < 0.001 for both). Nevertheless, there was no significant difference between any groups without or using different CoCl_2_ concentrations (*p* ≥ 0.215)

PAX6 mRNA and protein levels were significantly lower after *PAX6* siRNA knockdown compared to control siRNA use (*p* < 0.001 for both). However, no significant PAX6 mRNA and protein level difference was observed between the groups, using different CoCl_2_ concentrations (*p* ≥ 0.215) (Fig. 5).

### mRNA and protein expression of hypoxia-related markers, anti-oxidative enzymes and inflammatory cytokines in control and PAX6 knockdown LECs after CoCl_**2**_ treatment

Due to the low HIF-1α and hypoxia-inducible factor 1-alpha (HIF-2α) protein levels in LECs, limbal stromal cells (LSCs) were used as positive controls for HIF-1α and HIF-2α protein level measurement using Western blot (Supplemental, Fig. 1).

**Fig. 6:**
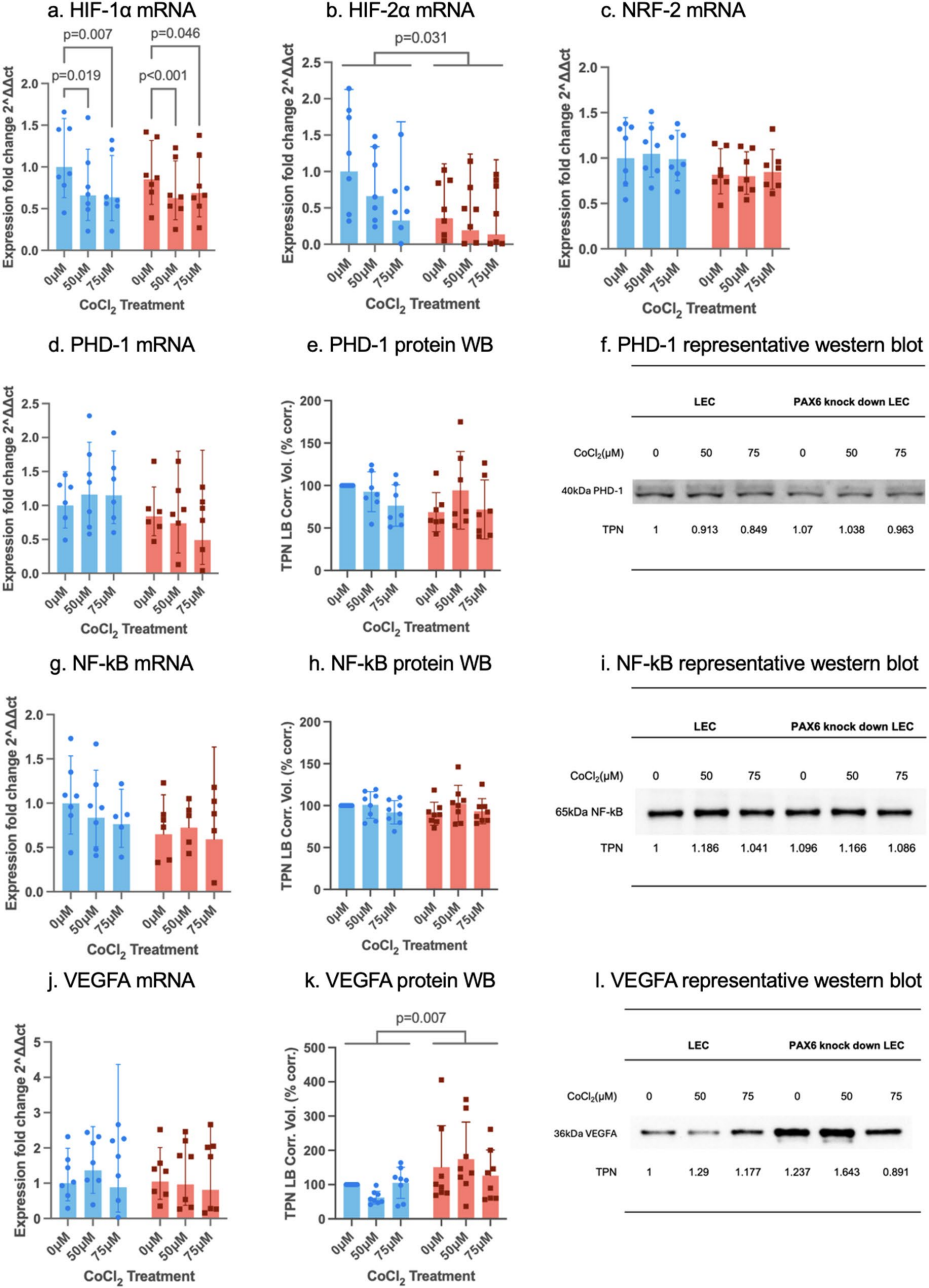
Hypoxia-Inducible Factor 1 Alpha (HIF-1α), Hypoxia-Inducible Factor 2 Alpha (HIF-2α), Nuclear Factor Erythroid 2-Related Factor 2 (NRF-2), Prolyl Hydroxylase Domain Containing Protein 1 (PHD-1), Nuclear Factor kappa B (NF-kB) and Vascular Endothelial Growth Factor A (VEGFA) mRNA and protein levels in control siRNA (blue bars) and in PAX6 knockdown (red bars) limbal epithelial cells (LECs) without and after 50µM or 75µM CoCl_2_ incubation for 48 h (**a-l**). Quantitative PCR (qPCR; *n* = 7 biological replicates, each with 2 technical replicates) and Western blotting (WB; *n* = 8 biological replicates, each with 1 technical replicate) were performed. qPCR, Western blot, and ELISA were performed using the same donor pool with assay-specific subsets depending on sample availability (Table 1). mRNA values are shown as geometric mean±geometric standard deviation (SD). Total protein staining was used for protein normalization of each lane. The total protein normalization factor (TPN) is indicated below each lane. Band intensity from WB is shown with mean ± SD. Two-way ANOVA, followed by Dunnett’s test was used. A repeated-measures design was used to account for donor pairing. Outliers were excluded using Grubbs’ test. Significant p values (< 0.05) are highlighted *HIF-2α* mRNA level was downregulated (**b**) and VEGFA protein level was upregulated (**k**) in *PAX6* knockdown LECs (*p* = 0.031, *p* = 0.007) 50 µM and 75 µM CoCl_2_ treatment downregulated *HIF-1α* mRNA expression in both control siRNA treated (*p* ≤ 0.019) and in *PAX6* knockdown LECs (*p* ≤ 0.046) (**a**). Nevertheless, there was no significant difference between any other groups without or using different CoCl_2_ concentrations (*p* ≥ 0.179)

Between hypoxia-related markers, *HIF-2α* mRNA levels were downregulated and VEGFA protein levels were upregulated in *PAX6* knockdown LECs (*p* = 0.031, *p* = 0.007). Treatment with 50 µM and 75 µM CoCl_2_ downregulated *HIF-1α* mRNA expression in both control siRNA-treated (*p* ≤ 0.019) and *PAX6* knockdown LECs (*p* ≤ 0.046) (Fig. 6). Nevertheless, NRF-2, PHD-1 and NF-kB mRNA and protein levels did not differ between groups (*p* ≥ 0.179) (Fig. 6).

**Fig. 7:**
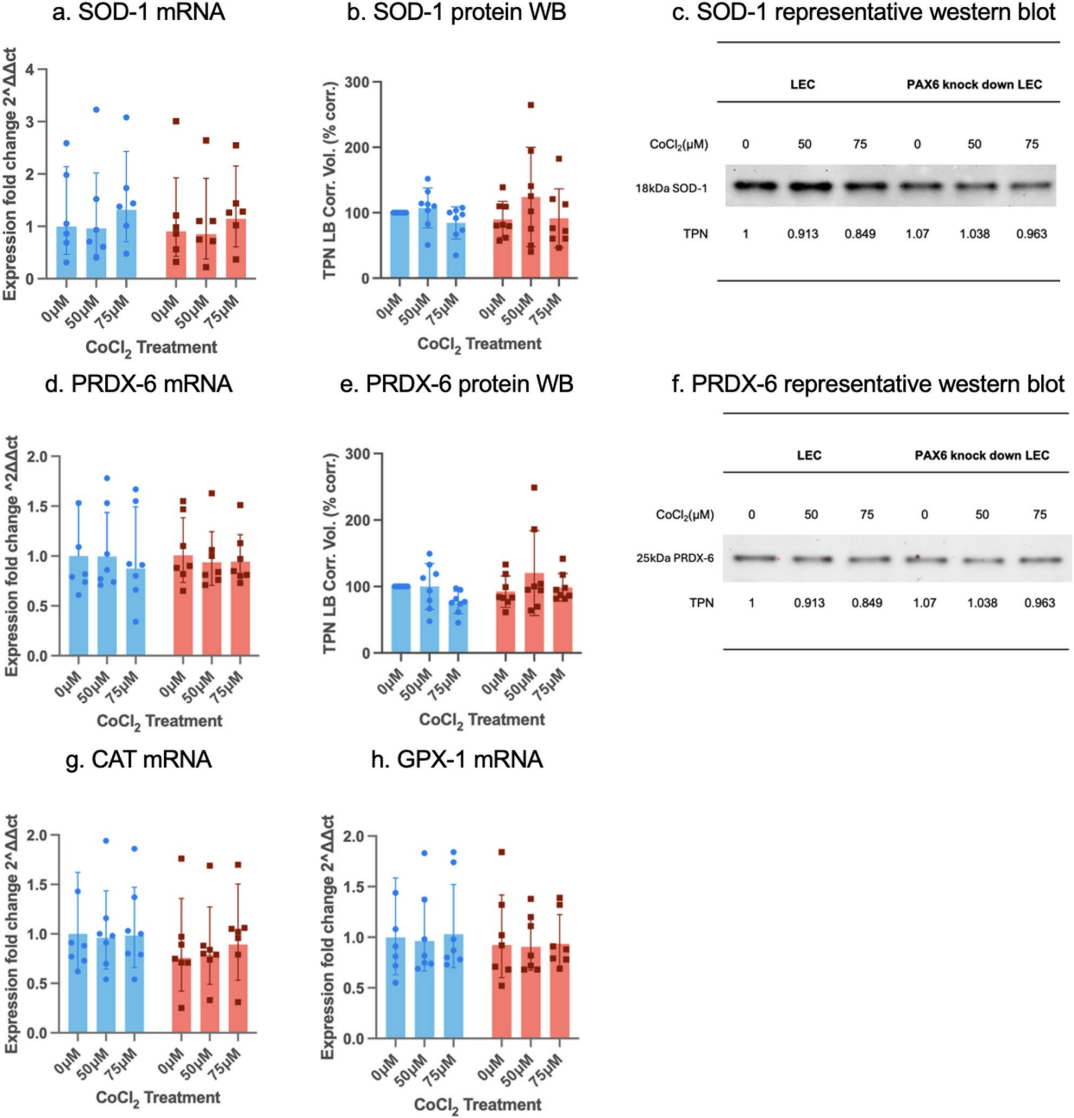
Superoxide Dismutase 1 (SOD-1), Peroxiredoxin 6 (PRDX-6), Catalase (CAT) and Glutathione Peroxidase 1 (GPX-1) mRNA and protein levels in control siRNA (blue bars) and in PAX6 knockdown (red bars) limbal epithelial cells (LECs) without and after 50µM or 75µM CoCl_2_ incubation for 48 h (**a-h**). Quantitative PCR (qPCR; *n* = 7 biological replicates, each with 2 technical replicates) and Western blotting (WB; *n* = 8 biological replicates, each with 1 technical replicate) were performed. qPCR, Western blot, and ELISA were performed using the same donor pool with assay-specific subsets depending on sample availability (Table 1). mRNA values are shown as geometric mean±geometric standard deviation (SD). Total protein staining was used for protein normalization of each lane. The total protein normalization factor (TPN) is indicated below each lane. Band intensity from WB is shown with mean ± SD. Two-way ANOVA, followed by Dunnett’s test was used. A repeated-measures design was used to account for donor pairing. Outliers were excluded using Grubbs’ test. Significant p values (< 0.05) are highlighted There was no significant difference between any of the groups with control siRNA treatment or *PAX6* knockdown, without or using different CoCl_2_ concentrations (*p* ≥ 0.159)

Between anti-oxidative enzymes, SOD-1, PRDX-6, CAT and GPX-1 mRNA and protein levels did not differ between controls siRNA and *PAX6* control group (*p* ≥ 0.159) (Fig. 7).

**Fig. 8:**
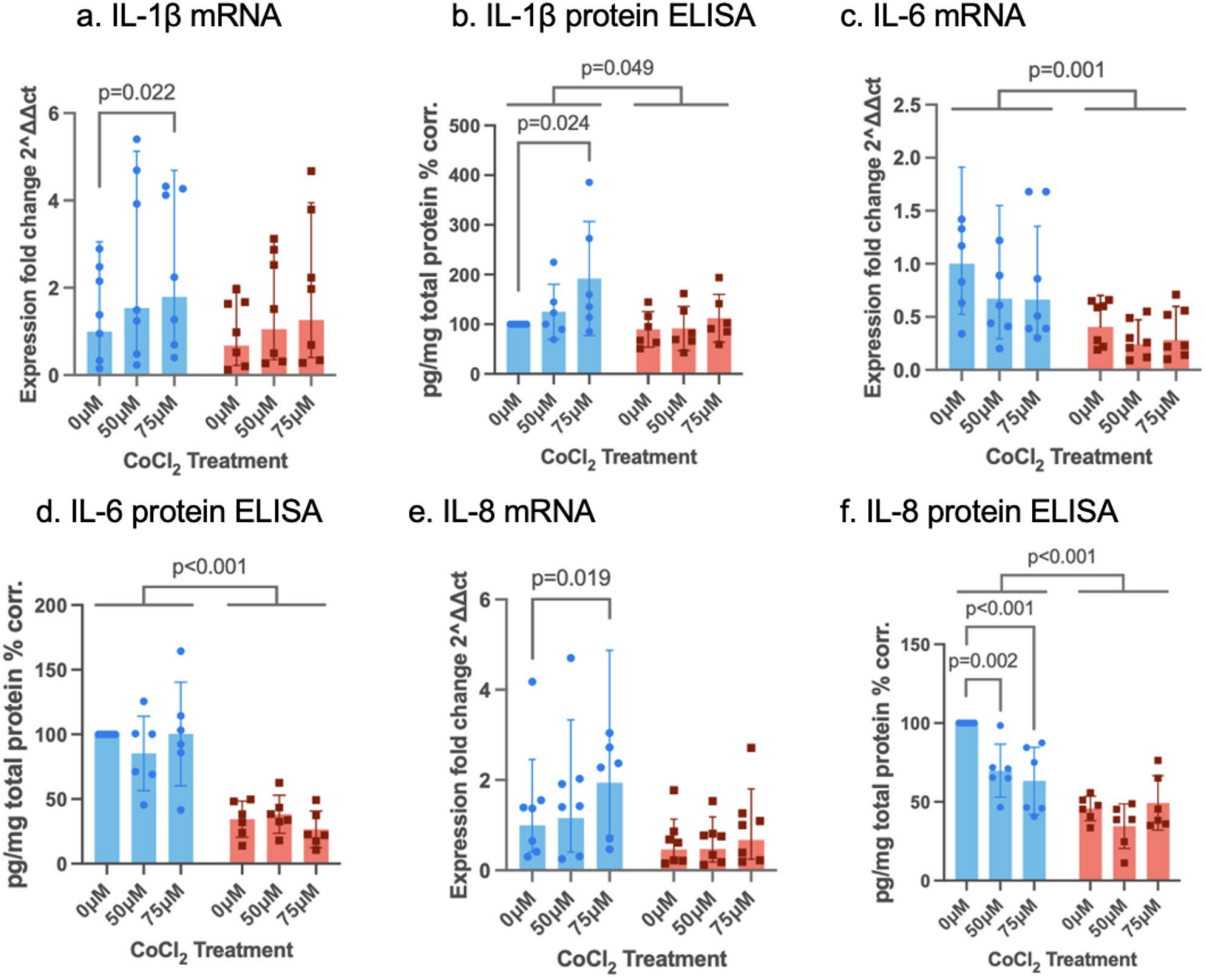
Interleukin 1 Beta (IL-1β), Interleukin 6 (IL-6) and Interleukin 8 (IL-8) mRNA and protein levels in control siRNA (blue bars) and in PAX6 knockdown (red bars) limbal epithelial cells (LECs) without and after 50µM or 75µM CoCl_2_ incubation for 48 h (**a-f**). Quantitative PCR (qPCR; *n* = 7 biological replicates, each with 2 technical replicates) and enzyme-linked immunosorbent assay (ELISA; *n* = 6 biological replicates, each with 2 technical replicates) were performed. qPCR, Western blot, and ELISA were performed using the same donor pool with assay-specific subsets depending on sample availability (Table 1). CT values are shown as geometric mean±geometric standard deviation (SD). Total protein staining was used for protein normalization of each lane. The total protein normalization factor (TPN) is indicated below each lane. Band intensity from WB is shown with mean ± SD. Two-way ANOVA, followed by Dunnett’s test was used. A repeated-measures design was used to account for donor pairing. Outliers were excluded using Grubbs’ test. Significant p values (< 0.05) are highlighted IL-1β protein level (**b**), *IL-6* mRNA (**c**), IL-6 protein (**d**) and IL-8 protein (**f**) levels were downregulated in *PAX6* knockdown LECs, compared to controls (*p* ≤ 0.049). In control siRNA-treated LECs, 50 µM CoCl_2_ resulted in a decrease in IL-8 protein levels compared to untreated controls (**f**) (*p* = 0.002). Furthermore, in control siRNA-treated LECs, treatment with 75 µM CoCl_2_ led to an increase in *IL-1β* (**a**) and *IL-8* (**e**) mRNA levels (*p* = 0.022, *p* = 0.019), IL-1β protein levels (**b**) (*p* = 0.024), while also causing a decrease in IL-8 protein levels (**f**) (*p* < 0.001)

Between inflammatory cytokines, IL-1β protein levels, *IL-6* mRNA, IL-6 protein, and IL-8 protein levels were significantly reduced in *PAX6* knockdown LECs compared to the control group (*p* ≤ 0.049). In control siRNA LECs, 50 µM CoCl_2_ led to reduced IL-8 protein level, compared to untreated controls (*p* = 0.002). In addition, in control siRNA-treated LECs, treatment with 75 µM CoCl_2_ led to an increase in *IL-1β* and *IL-8* mRNA levels (*p* = 0.022, *p* = 0.019), IL-1β protein levels (*p* = 0.024), as well as to a decrease of IL-8 protein level (*p* < 0.001) (Fig. 8).

## Discussion

In healthy cells, increased oxidative stress activates transcription factors such as HIF-1α, NF-κB, and NRF-2, leading to the upregulation of hypoxia-related markers, inflammatory cytokines, and antioxidative enzymes. However, the impact of CoCl₂-induced stress on *PAX6*-haploinsufficient LECs remains poorly understood.

In vitro hypoxia can be modeled either by culturing cells under reduced oxygen tension or by using chemical hypoxia mimetics such as CoCl₂. While low-oxygen culture conditions more closely resemble physiological hypoxia, they are associated with several technical limitations, including delayed stabilization of oxygen levels within covered culture plates and increased experimental variability [17]. In the context of the present study, these constraints are further compounded by the limited experimental window imposed by siRNA transfection, which restricts the feasibility of prolonged or tightly controlled low-O₂ exposure. Therefore, CoCl₂ treatment used as a hypoxia-mimetic stressor was selected as a reproducible alternative approach in the present study.

However, the results should therefore be interpreted as responses under CoCl₂ treatment rather than a true physiological hypoxic condition. Although this hypoxia-mimetic approach has been validated in our previous fibroblast studies and used here for methodological consistency [18], its limitations in LECs are acknowledged. In particular, HIF-1α and HIF-2α protein levels in LECs were below the detection limit of Western blot analysis (Supplementary Fig. 1), and the expected hypoxia-induced upregulation of *HIF-1α* mRNA was not observed. Therefore, it cannot be excluded that the effects reflect CoCl₂-induced cellular toxicity or a combined effect rather than a validated hypoxic response. Accordingly, we suggest the interpretation of the observed responses with caution and should only be restricted to the present model under the given experimental constraints instead of over-interpreting as mechanistic evidence of hypoxia-related pathways.

Several changes in primary aniridia limbal fibroblast cells (AN-LFCs) under hypoxia-mimetic CoCl₂ exposure have been described in a previous study [18]. AN-LFCs exhibited significantly decreased *PHD-1* mRNA levels in response to CoCl₂-induced stress, and *HIF-1α* was observed to be downregulated under 75µM CoCl_2_ treatment in both normal LFCs and PAX6-knockdown LFCs, whereas *HIF-2α* showed no significant changes [18].

Although HIF-1α is expected to be upregulated as the main regulator in response to hypoxic conditions in many cases [19], our results suggest a significantly reduced mRNA expression level of *HIF-1α* with increased CoCl₂-induced stress in *PAX6* knockdown LECs. In addition to the limitation that CoCl₂-induced conditions may not fully replicate physiological hypoxia, this finding may reflect negative feedback regulation of *HIF-1α* under chronic stress as part of cellular adaptive responses [20]. This feedback mechanism may help prevent prolonged damage, including angiogenesis, cell remodeling, and inflammatory responses, which could result from excessive activation of HIF-1α. In addition to oxygen-dependent regulation mediated by PHD-1, HIF-1α expression can also be regulated by oxygen-independent mechanisms, including post-transcriptional and post-translational modifications [21].

In real-world conditions, HIF-1α and HIF-2α may not be tightly coupled in their responses to hypoxic stress. HIF-1α primarily mediates cellular responses to acute hypoxia and is tightly regulated via the PHD-1 pathway, whereas HIF-2α regulates overlapping but distinct target genes in a more context-dependent manner, contributing predominantly to long-term or chronic hypoxia adaptation depending on cell type and microenvironmental cues [22, 23]. Although HIF-1α is a well-established regulator of VEGFA expression, our data do not demonstrate a direct correlation between HIF-1α and VEGFA levels in this model. VEGFA is clinically relevant and may reflect pathological processes associated with aniridia-associated keratopathy, including corneal neovascularization and impaired epithelial wound healing [7].

In the present study, VEGFA protein expression was increased following *PAX6* knockdown in limbal epithelial cells, suggesting a potential link between *PAX6* haploinsufficiency and angiogenesis-related signaling. PAX6 may influence angiogenic processes through both direct and indirect mechanisms. Directly, PAX6 may regulate corneal angiogenesis via soluble vascular endothelial growth factor receptor-1 (sVEGFR-1)–mediated VEGF sequestration [24]. Indirectly, PAX6 is essential for maintaining limbal epithelial stem cell homeostasis, and its loss may disrupt the limbal niche, thereby promoting a pro-angiogenic microenvironment and aberrant vascular ingrowth [25].

Previous studies have suggested that epithelial cells exhibit a relatively limited response to CoCl₂-induced stress, whereas corneal fibroblasts display more pronounced alterations in oxidative stress– and inflammation-related markers under CoCl₂ treatment [26]. Our previous study of AN-LFCs shows altered expression of *NRF-2* and its regulated antioxidative enzymes compared to normal LFCs [18]. However, there is no significant change of NF-κB, *NRF-2*, and its related downstream antioxidant enzymes observed in our experiment, using *PAX6* haploinsufficient LECs. Rather than dependence on different cell types [27], another possibility for the lack of change in the expression of these transcription factors like NF-κB, and NRF-2 could be due to independent regulation from PAX6 or CoCl_2_ treatment. For example, NF-κB activation is primarily regulated by the canonical pathway involving the IKK complex activation and degradation of its inhibitor IκB. However, NF-κB can also be regulated through multiple signaling pathways, including non-canonical NF-κB pathways, which might be less sensitive to changes in PAX6 expression or CoCl_2_-induced stress [28]. This ensures that transcription factors maintaining critical physiological functions in cells are not easily affected by environmental changes, preventing significant fluctuations in gene expression.

NRF-2 serves as a master transcriptional regulator of antioxidant response genes, including GPX-1, PRDX-6, CAT, and SOD-1 [12, 29]. Its activation is typically triggered by ROS-related oxidative stress rather than directly by hypoxic conditions [12]. Only severe hypoxia conditions lead to ROS accumulation, resulting in oxidative stress within the cell. As a result, the expression of NRF-2 and its target genes may be dose-dependent, where the concentration and duration of CoCl_2_ treatment in our experiment may not have reached the threshold required to significantly induce the NRF-2-mediated downstream response.

Our experiments show altered inflammatory signaling in *PAX6* knockdown LECs, with reduced baseline expression of IL-1β, IL-6, and IL-8 or a diminished response to CoCl₂-induced stress compared with controls, suggesting impaired inflammatory regulation in the absence of PAX6 (Fig. 8). Dysregulation of proinflammatory cytokines from the ocular surface is also evidenced in aniridia subjects with AAK [30]. Inflammation under hypoxic stress can be a double-edged sword, the study from Zhang JM et al. suggests increased secretory inflammatory cytokines potentially benefit cell-cell communication, and also promote tissue repair and regeneration through cell migration and proliferation [31]. This natural protective mechanism is strongly associated with HIF pathway activation to promote antioxidant defense [32], which may relate to the poor healing response observed in aniridia patients with AAK [33].

In summary, our study reveals alterations in the regulation of pro-inflammatory cytokine secretion and its associated HIF-mediated pathway, but unchanged *HIF-1α*, NF-κB, and *NRF-2* levels in in *PAX6* knockdown LECs compared to healthy LECs following CoCl_2−_induced stress. While earlier research primarily focused on individual cell types, recent studies have shifted attention toward the limbal stem cell niche [14] and the interaction between LECs and LFCs to better explain the context of AAK [34]. Our results further indicate that both *PAX6* knockdown limbal epithelial cells and aniridia-derived limbal fibroblasts exhibit altered regulatory responses to hypoxia-mimetic CoCl₂ exposure compared with normal limbal cells, which may help bridge the characteristic epithelial and stromal changes observed in AAK [18].

This study has several limitations. First, transcription factors and their targets may show transient or delayed responses to stimuli, which may not be fully captured in short-term experiments [35]. Longer-term studies or additional stressors may better reveal dynamic gene regulation [35]. Second, while we used siRNA-mediated *PAX6* knockdown to mimic *PAX6* haploinsufficiency, congenital aniridia can also result from broader deletions involving neighboring genes such as Wilms’ tumor 1 (WT1) [6]. Thus, our model may not fully replicate the disease’s complexity but provides insight into the effects of reduced PAX6 under hypoxia-mimetic CoCl₂ conditions [36]. Furthermore, native aniridia LECs are more susceptible to oxidative stress than siRNA knockdown cells [36]. Therefore, the healthy donor-derived primary limbal epithelial cells with transient *PAX6* knockdown used in this study do not fully recapitulate the alterations observed in aniridia-associated keratopathy. Importantly, the findings reflect cellular responses to CoCl₂-induced stress and may not fully represent a validated hypoxia model in LECs. Accordingly, additional markers such as HIF-1α and glucose transporter (GLUT1) should be assessed at the protein level in future studies to further validate the hypoxia-mimetic model. In addition, monocultured limbal epithelial cells lack the complex cellular interactions and niche-specific signals present in vivo, which may oversimplify the disease context. Moreover, hypoxia-associated gene expression is governed by intricate regulatory networks, and alterations in a single factor cannot reliably predict changes in others, as gene expression is shaped by multilayered regulatory crosstalk [37].

## Conclusions

In summary, this study demonstrates altered expression of *HIF-2α*, VEGFA, and selected pro-inflammatory cytokines at the mRNA and/or protein level in *PAX6* knockdown LECs compared with control siRNA-treated cells, while *HIF-1α*, NF-κB, and *NRF-2* levels remained unchanged.

Importantly, these findings reflect associations observed in a simplified *PAX6* knockdown model following CoCl₂-induced stress under defined in vitro conditions and should not be interpreted as definitive mechanistic evidence for aniridia-associated keratopathy. Therefore, more rigorous hypoxia models and the use of patient-derived in vivo systems are needed to further elucidate the pathogenesis of oxidative intolerance in AAK.

## Supplementary Information

Below is the link to the electronic supplementary material.

Supplementary Material 1: Representative hypoxia-inducible factor 1α (HIF-1α) (a) and hypoxia-inducible factor 2α (HIF-2α) (b) Western blots of limbal epithelial cells (LECs), paired box 6 (PAX6) knockdown LECs and limbal fibroblast cells (LFCs) as positive controls.

## Data Availability

The datasets of the study are available from the corresponding author upon reasonable request.
