# Supplementary material for: CoCl2-induced alterations in antioxidative and inflammatory marker expression in an siRNA-based in vitro model of aniridia-associated limbal epithelial dysfunction
Source: BMC Ophthalmol. 2026 Jun 6;26:316. doi: 10.1186/s12886-026-04982-8 (PMC13244889; doi:10.1186/s12886-026-04982-8)

**CoCl_2_-Induced Alterations in Antioxidative and Inflammatory Marker Expression in an siRNA-Based *In Vitro* Model of Aniridia-Associated Limbal Epithelial Dysfunction**

Shao-Lun Hsu ^1, 2^, Nóra Szentmáry ^1, 2^, Fabian N. Fries ^1, 3^, Zhen Li ^1^, Ning Chai ^1^, Berthold Seitz ^3^, Maryam Amini ^1^, Shweta Suiwal ^1, 2^, Tanja Stachon ^1, 2^

^1^ Dr. Rolf M. Schwiete Center for Limbal Stem Cell and Congenital Aniridia Research, Saarland University, Homburg/Saar, Germany

^2^ Experimental Ophthalmology, Saarland University, Homburg/Saar, Germany

^3^ Department of Ophthalmology, Saarland University Medical Center, Homburg/Saar, Germany

| 1. HIF-1α representative western blot | 1. HIF-2α representative western blot |
| --- | --- |
| 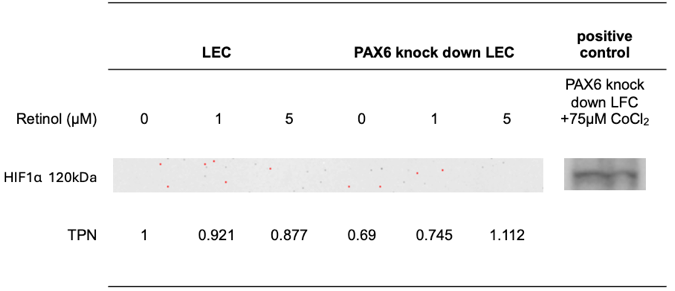 | 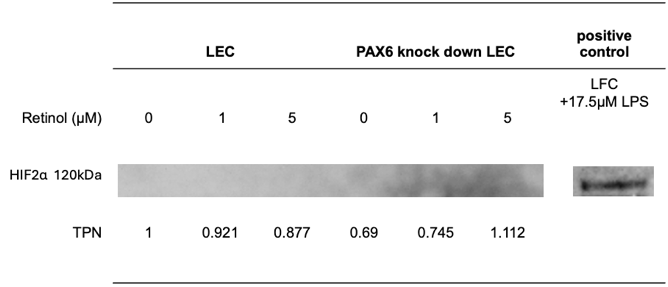 |

**Supplementary figure 1. Representative hypoxia-inducible factor 1α (HIF-1α) (a) and hypoxia-inducible factor 2α (HIF-2α) (b) Western blots of limbal epithelial cells (LECs),** ***paired box 6* (*PAX6*) knockdown LECs and limbal fibroblast cells (LFCs)**  **as positive controls.** There are low HIF-1α (a) and HIF-2α (b) protein levels as representative Western blots of cell lysates of LECs. LFCs followig 75 µM CoCl_2_ and 17.5 µM lipopolysaccharide (LPS) treatment has been used as positive controls. Western blotting was performed using n=8 biological replicates (1 technical replicate each). Total protein staining was used for normalization of each lane, and the total protein normalization (TPN) factor is indicated below each lane.

**The original uncropped figure.** The full-length original, unprocessed versions of all blots are provided below, following the order in the manuscript. Please note the coding system shown in figure:

| X.1.1 | donor number X, *PAX6* control LEC+0µM CoCl_2_ |
| --- | --- |
| X.1.2 | donor number X, *PAX6* control LEC+50µM CoCl_2_ |
| X.1.3 | donor number X, *PAX6* control LEC+75µM CoCl_2_ |
| X.2.1 | donor number X, *PAX6* knockdown LEC+0µM CoCl_2_ |
| X.2.2 | donor number X, *PAX6* knockdown LEC+50µM CoCl_2_ |
| X.2.3 | donor number X, *PAX6* knockdown LEC+75µM CoCl_2_ |

**Figure 5c.** PAX6 representative western blot


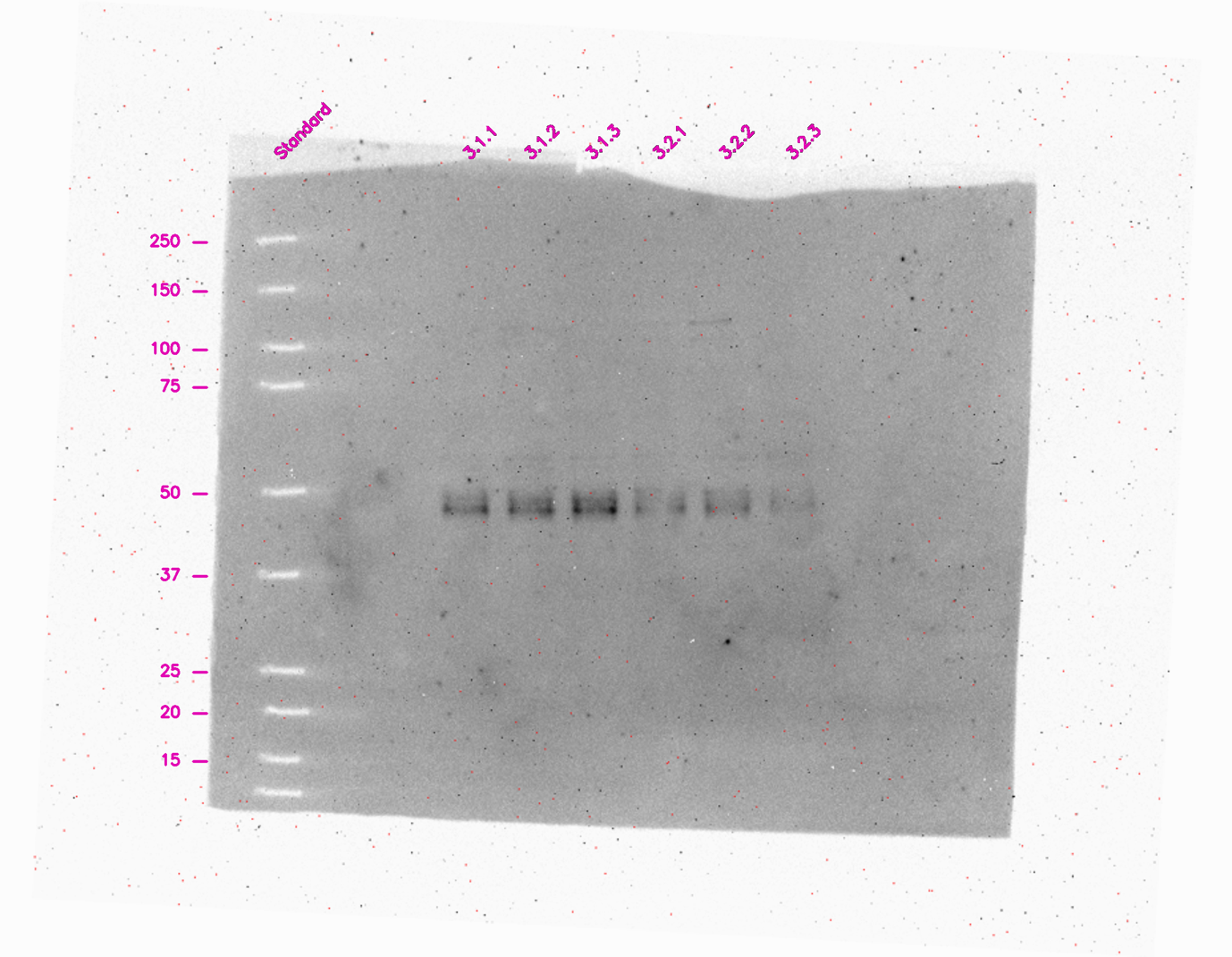


**Figure 6f.** PHD-1representative western blot


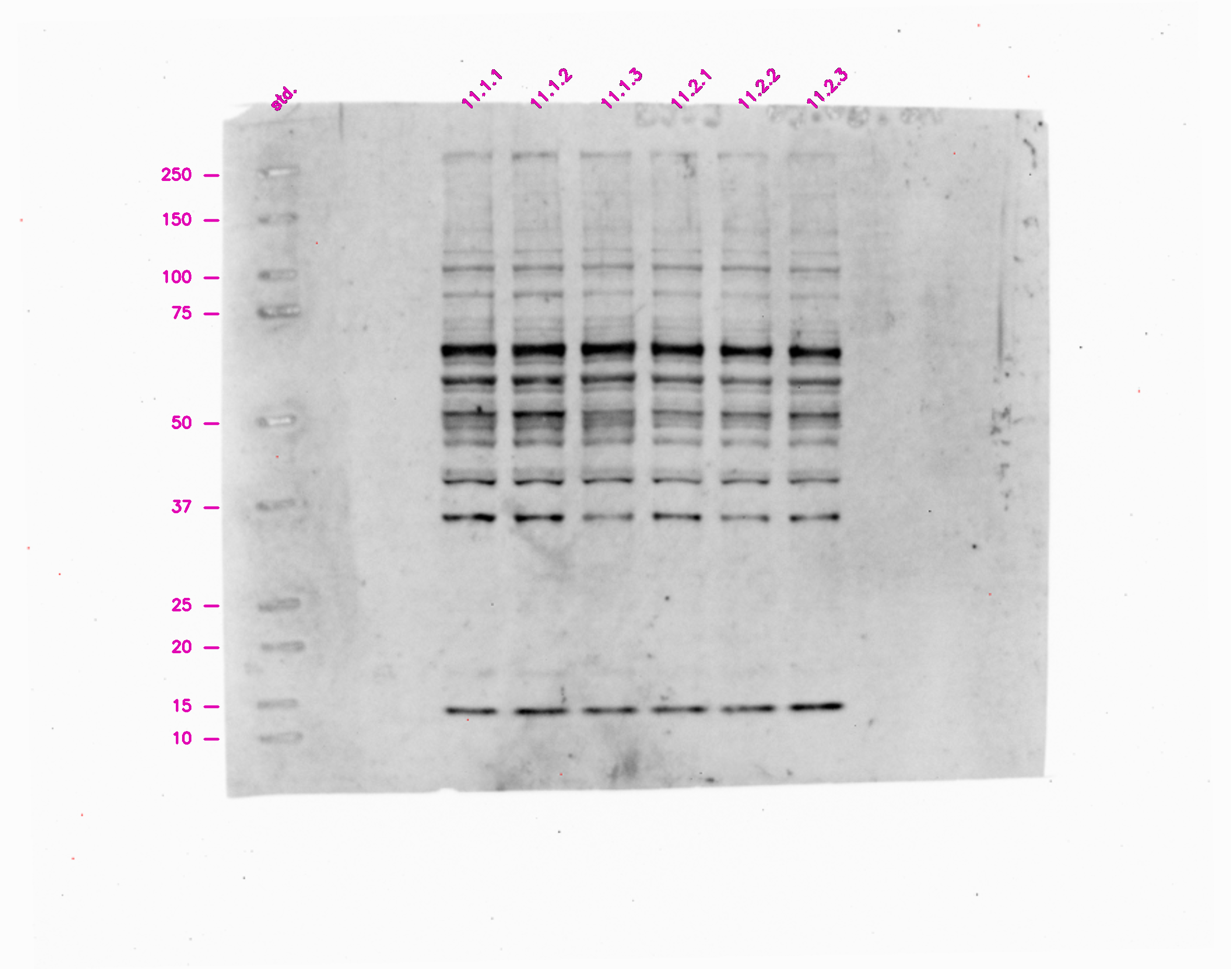


**Figure 6i.** NF-kB representative western blot


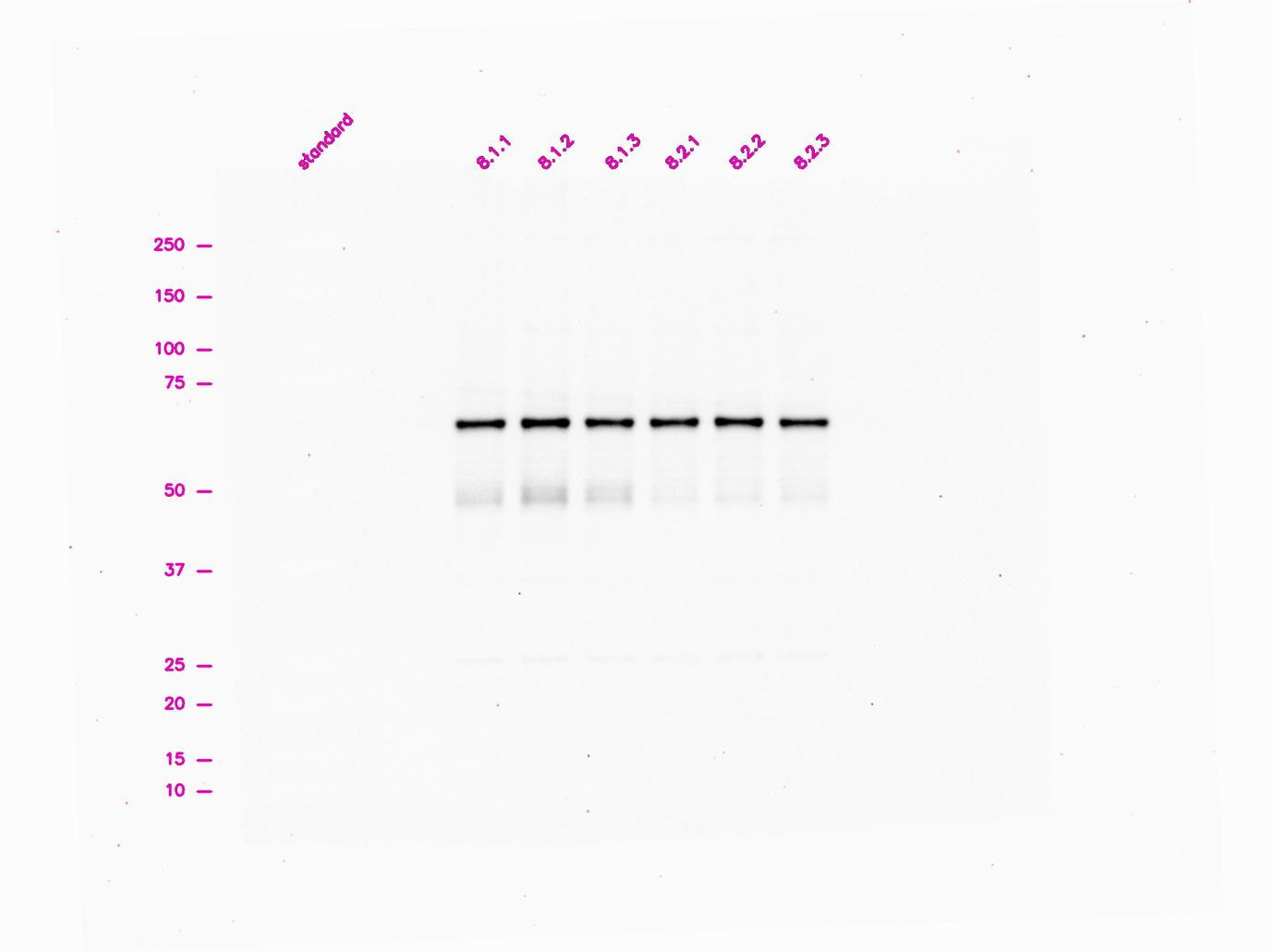


**Figure 6l.** VEGFA representative western blot


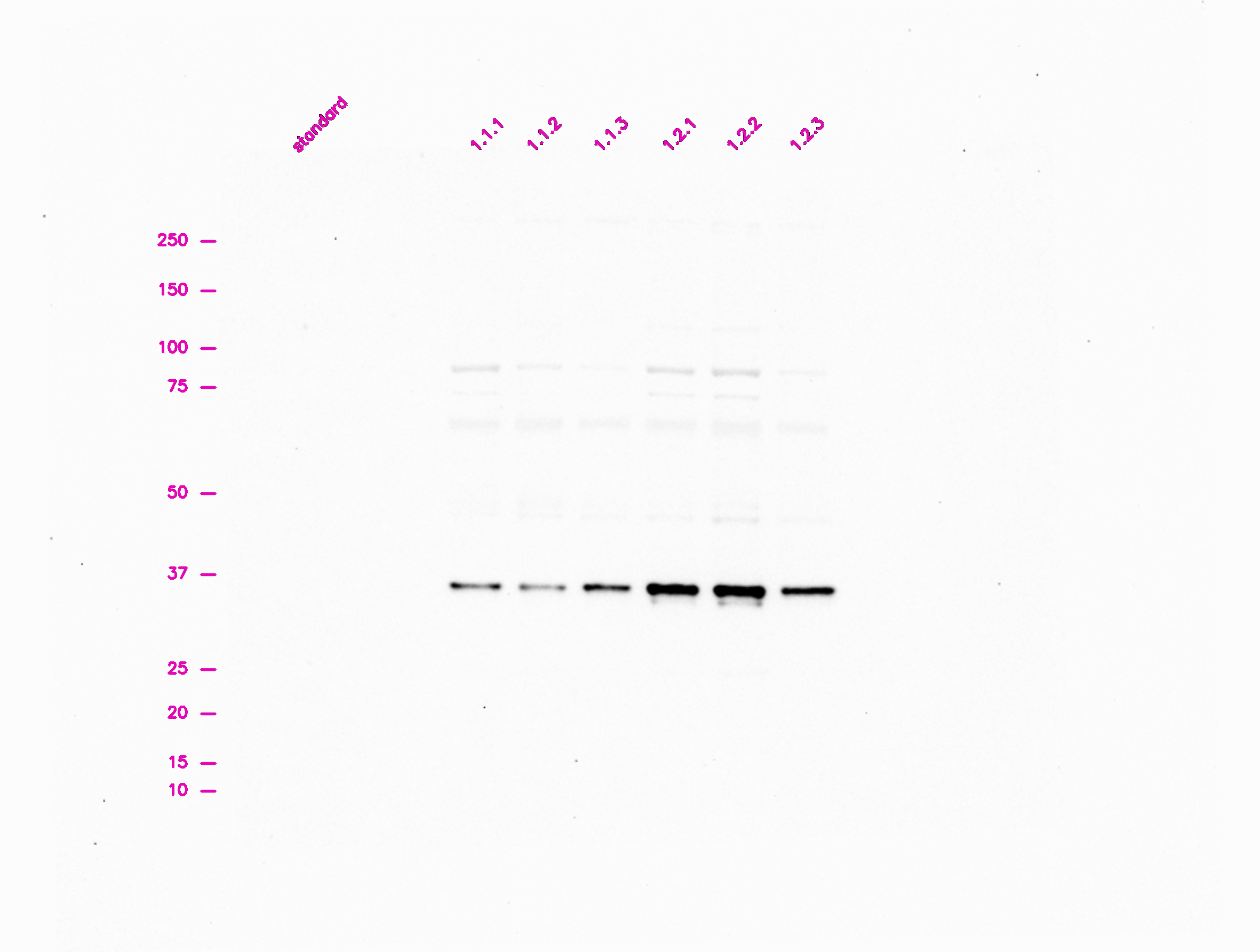


**Figure 7c.** SOD-1 representative western blot


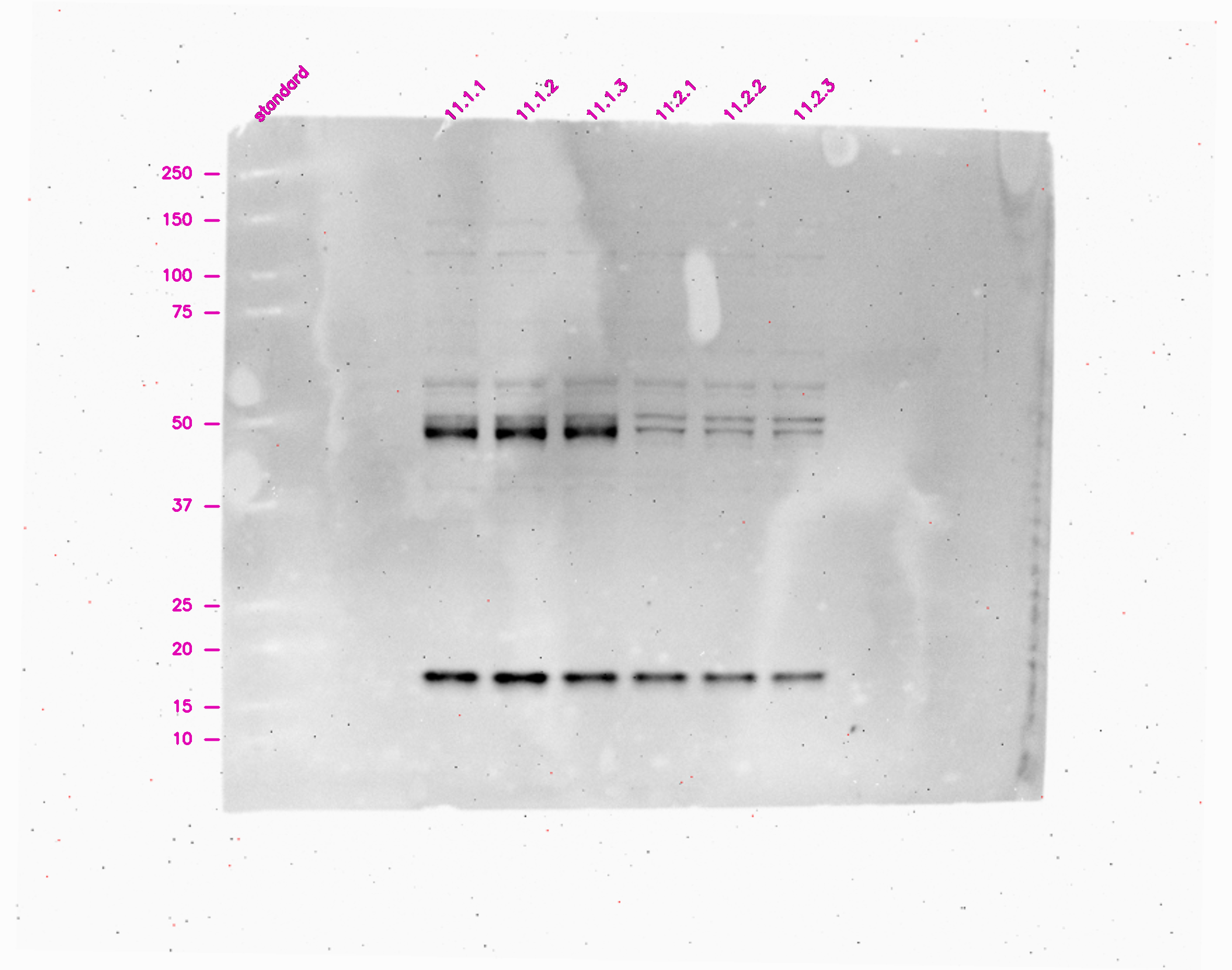


**Figure 7f.** PRDX-6 representative western blot


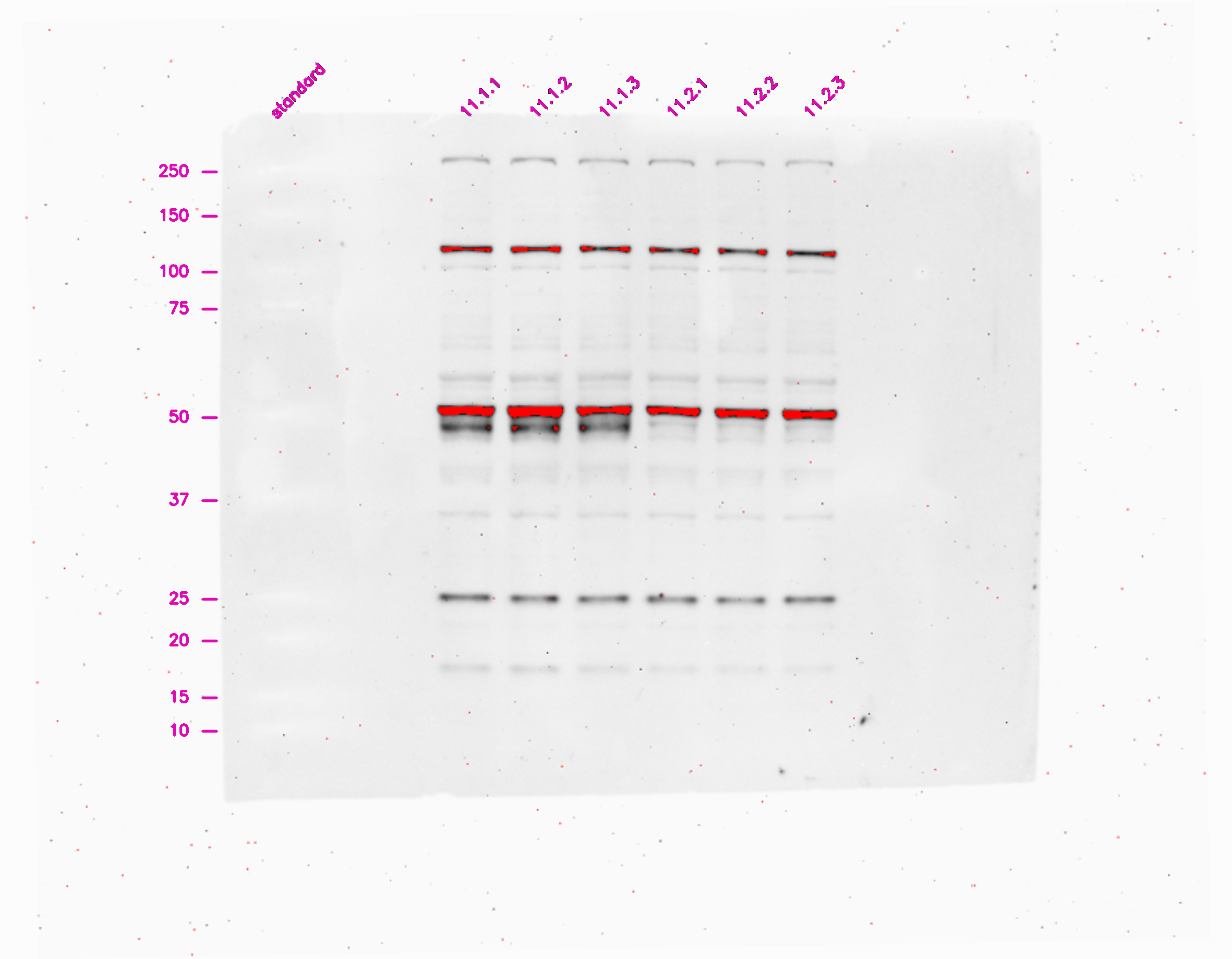


**Supplementary figure 1a.** HIF-1α representative western blot


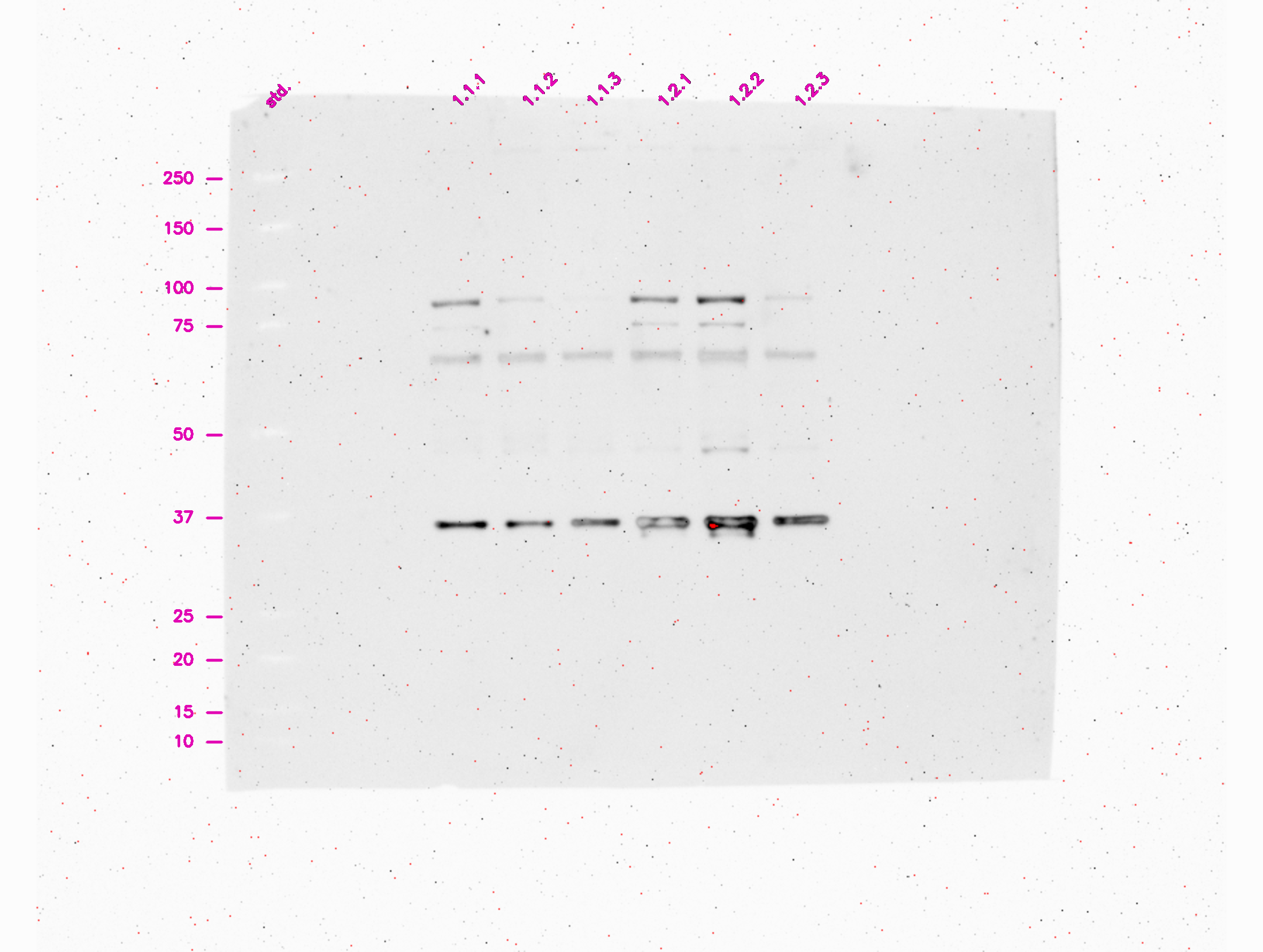


**Supplementary figure 1a.** HIF-1α positive control


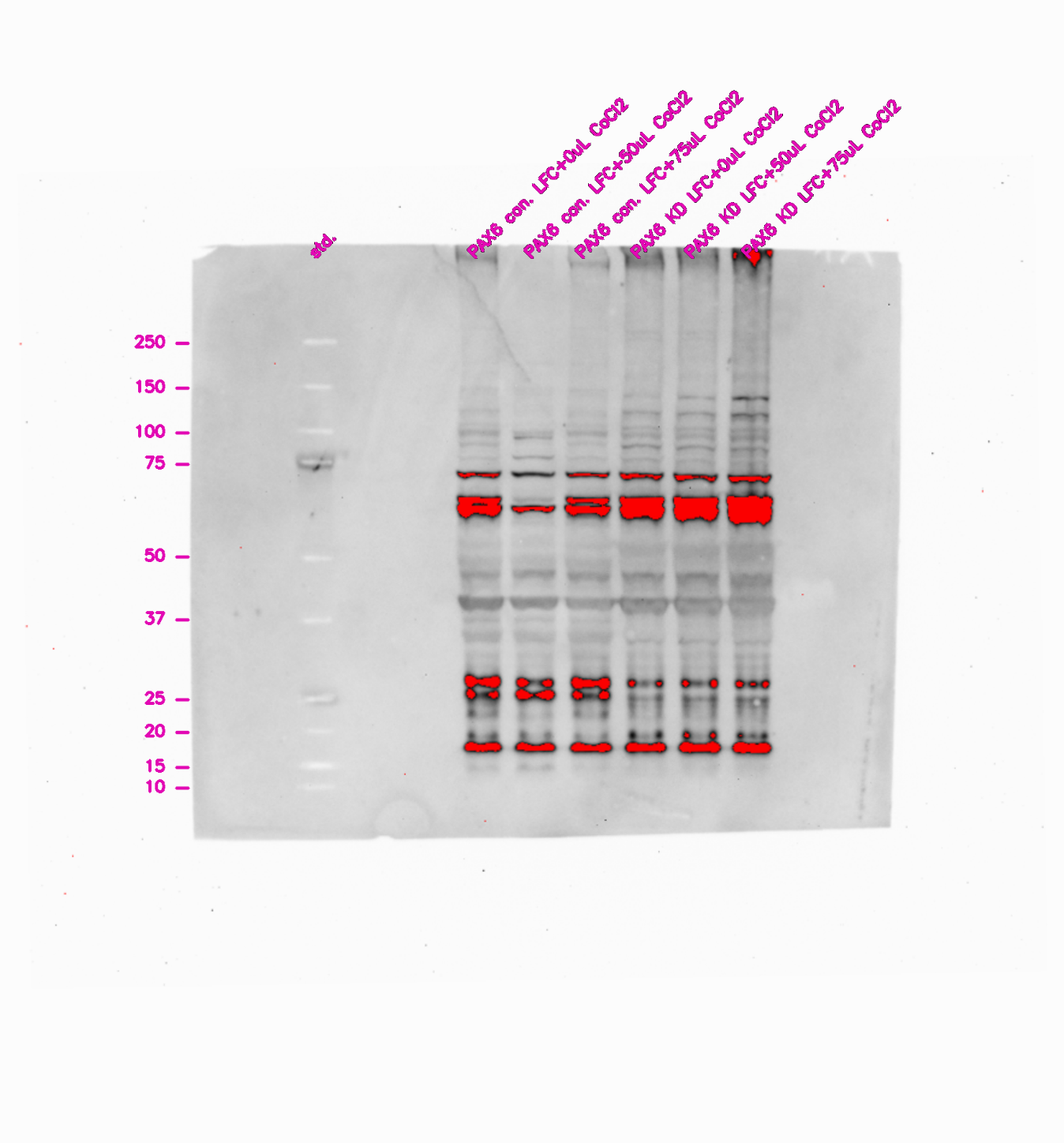


**Supplementary figure 2α.** HIF-2α representative western blot


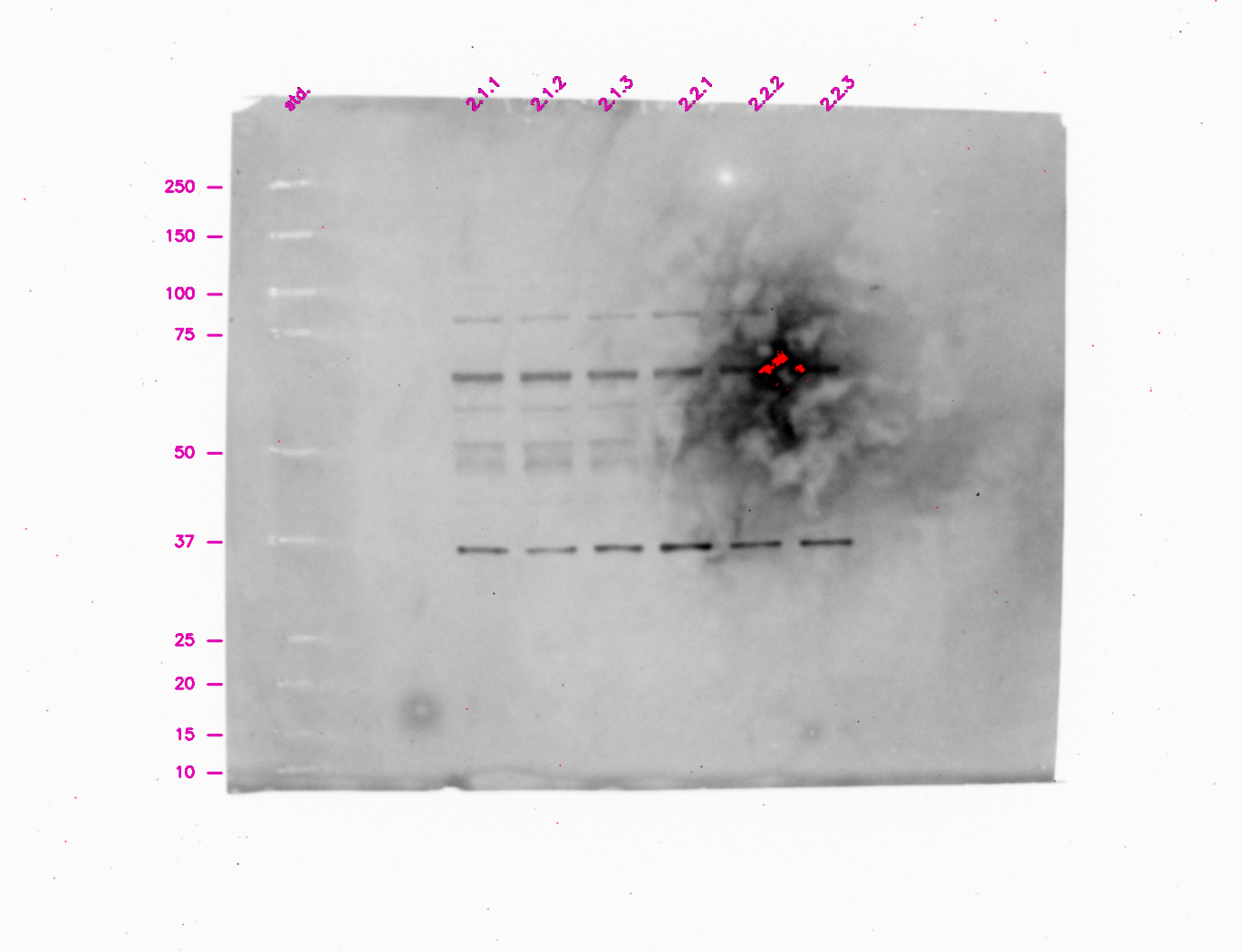


**Supplementary figure 2α.** HIF-2α positive control


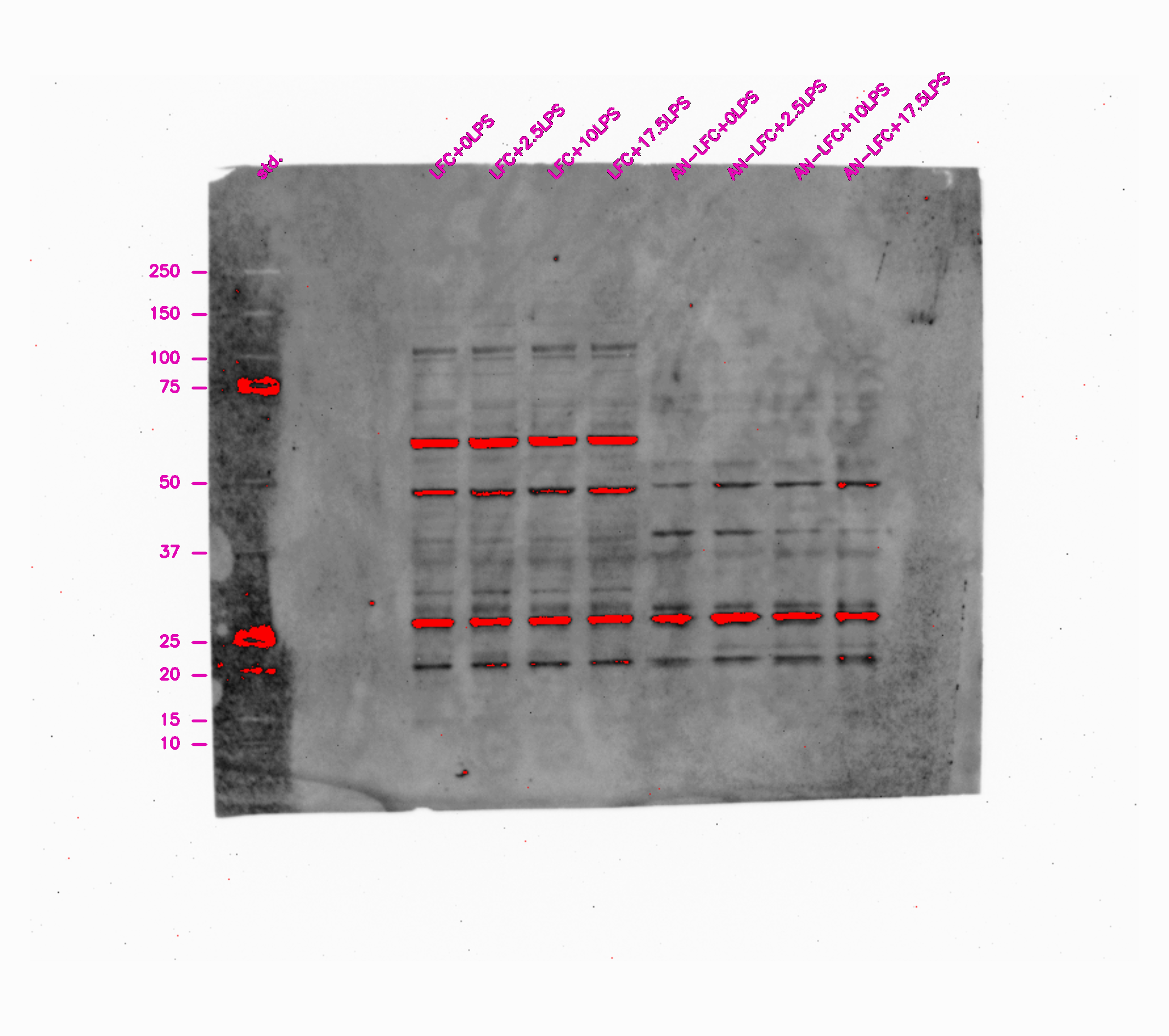

Supplement: Supplementary file 1 — Supplementary Material 1: Representative hypoxia-inducible factor 1α (HIF-1α) (a) and hypoxia-inducible factor 2α (HIF-2α) (b) Western blots of limbal epithelial cells (LECs), paired box 6 (PAX6) knockdown LECs and limbal fibroblast cells (LFCs) as positive controls. [file 12886_2026_4982_MOESM1_ESM.docx]
